# A new ankylosaurine dinosaur from the Judith River Formation of Montana, USA, based on an exceptional skeleton with soft tissue preservation

**DOI:** 10.1098/rsos.161086

**Published:** 2017-05-10

**Authors:** Victoria M. Arbour, David C. Evans

**Affiliations:** 1 Department of Palaeobiology, Royal Ontario Museum, 100 Queen's Park, Toronto, Ontario, Canada, M5S 2C6; 2 Department of Ecology and Evolutionary Biology, University of Toronto, 25 Willcocks Street, Toronto, Ontario, CanadaM5S 3B2

**Keywords:** Ornithischia, Thyreophora, Ankylosauria, Ankylosaurinae, Cretaceous, Campanian

## Abstract

The terrestrial Judith River Formation of northern Montana was deposited over an approximately 4 Myr interval during the Campanian (Late Cretaceous). Despite having been prospected and collected continuously by palaeontologists for over a century, few relatively complete dinosaur skeletons have been recovered from this unit to date. Here we describe a new genus and species of ankylosaurine dinosaur, *Zuul crurivastator*, from the Coal Ridge Member of the Judith River Formation, based on an exceptionally complete and well-preserved skeleton (ROM 75860). This is the first ankylosaurin skeleton known with a complete skull and tail club, and it is the most complete ankylosaurid ever found in North America. The presence of abundant soft tissue preservation across the skeleton, including *in situ* osteoderms, skin impressions and dark films that probably represent preserved keratin, make this exceptional skeleton an important reference for understanding the evolution of dermal and epidermal structures in this clade. Phylogenetic analysis recovers *Zuul* as an ankylosaurin ankylosaurid within a clade of *Dyoplosaurus* and *Scolosaurus*, with *Euoplocephalus* being more distantly related within Ankylosaurini. The occurrence of *Z. crurivastator* from the upper Judith River Formation fills a gap in the ankylosaurine stratigraphic and geographical record in North America, and further highlights that Campanian ankylosaurines were undergoing rapid evolution and stratigraphic succession of taxa as observed for Laramidian ceratopsids, hadrosaurids, pachycephalosaurids and tyrannosaurids.

## 1. Introduction

The Judith River Formation of northern Montana is a terrestrial siliciclastic unit deposited over an approximately 4 Myr interval during a Campanian transgressive-regressive cycle of the Western Interior Seaway [1]. The fossiliferous strata of the formation have been known to scientists since the Lewis and Clark expedition of the early 1800s, and the first vertebrate fossils from this unit were collected several decades later by F. V. Hayden, when mapping the area for the US Geological Survey [2]. This collection is historically significant because it included specimens recognized as the first dinosaurs from North America [3]. The Judith River Formation has been prospected and collected continuously by palaeontologists for over a century, but beyond abundant vertebrate microfossils [1–6], few relatively complete skulls or skeletons of dinosaurs or other vertebrates have been recovered. As such, dinosaur biodiversity in the Judith River Formation remains represented primarily by fragmentary specimens that are difficult to identify to low taxonomic levels. Only seven ornithischian dinosaur species that are currently considered valid have been identified from this unit, with most based on highly incomplete remains, and theropod species-level records are largely restricted to isolated teeth [7]. Knowledge of other aspects of the fauna, such as non-dinosaurian reptiles, is also based on isolated and fragmentary material.

The Oldman and Dinosaur Park formations of southern Alberta were deposited penecontemporaneously and under similar geological controls and palaeoenvironmental conditions as the Judith River Formation [8]. In contrast to the Judith River Formation, the Dinosaur Park Formation is taxonomically diverse. Over 45 dinosaur species are currently recognized from the Dinosaur Park Formation [9,10], of which the majority are large-bodied ornithischians (six hadrosaurids, ten ceratopsids and six ankylosaurians). The paucity of diagnostic fossil material in the Judith River Formation is particularly vexing given its significance in ongoing debates on dinosaur evolution and palaeobiogeography. The scant dinosaur remains from the Judith River Formation have contributed to discussions of provinciality [11–13], evolutionary mode and anagenesis [14,15] and faunal turnover [7]. New fossils from this unit, therefore, have considerable potential to provide important new data on these and other topics.

Of the common large-bodied plant-eating dinosaurs from the Campanian–Maastrichtian of North America, ankylosaurians are particularly poorly known from the Judith River Formation, being represented primarily by isolated teeth and bones [2,4,6,7]. Here we describe the first diagnostic ankylosaurid skeleton from the formation, representing the new ankylosaurine genus and species, *Zuul crurivastator*, based on a spectacular, partially mummified specimen with preserved soft tissues. The new specimen (ROM 75860) is one of the best dinosaur skeletons from this unit, and it is the most complete ankylosaurid ever found in North America, making it a key reference skeleton for interpreting more fragmentary specimens. Notably, it preserves both a complete skull and tail club, two of the most taxonomically informative parts of the ankylosaurine skeleton, yet which are rarely preserved in the same skeleton. ROM 75860 also has remarkable integument preservation across the body, including *in situ* osteoderms, skin impressions and dark films that probably represent preserved keratin. These features make the holotype of *Z. crurivastator* a critical specimen for interpreting other Laramidian ankylosaurine remains given that osteoderms typically dissociate from the rest of the skeleton after death, and will be important for understanding the evolution of dermal and epidermal structures in these unusual armoured vertebrates.

### 1.1. Institutional abbreviations

AMNH—American Museum of Natural History, New York, New York, USA; CMN—Canadian Museum of Nature, Ottawa, Ontario, Canada; MACN Pv—Colección nacional de Paleontología de Vertebrados del Museo Argentino de Ciencias Naturales ‘Bernardino Rivadavia’, Buenos Aires, Argentina; MOR—Museum of the Rockies, Bozeman, Montana, USA; MPC—Paleontological Center, Mongolian Academy of Sciences, Ulaanbaatar, Mongolia; NHMUK—The Natural History Museum, London, England, United Kingdom; PIN—Palaeontological Institute, Russian Academy of Sciences, Moscow, Russia; ROM—Royal Ontario Museum, Toronto, Ontario, Canada; TMP—Royal Tyrrell Museum of Palaeontology, Drumheller, Alberta, Canada; UALVP—University of Alberta Laboratory for Vertebrate Paleontology, Edmonton, Alberta, Canada; UMNH VP—Natural History Museum of Utah, Salt Lake City, Utah, USA; USNM—Smithsonian Museum of Natural History, Washington, DC, USA; ZPAL—Zakład Paleobiologii, Polish Academy of Sciences, Warsaw, Poland.

## 2. Material and methods

**Figure 1.**
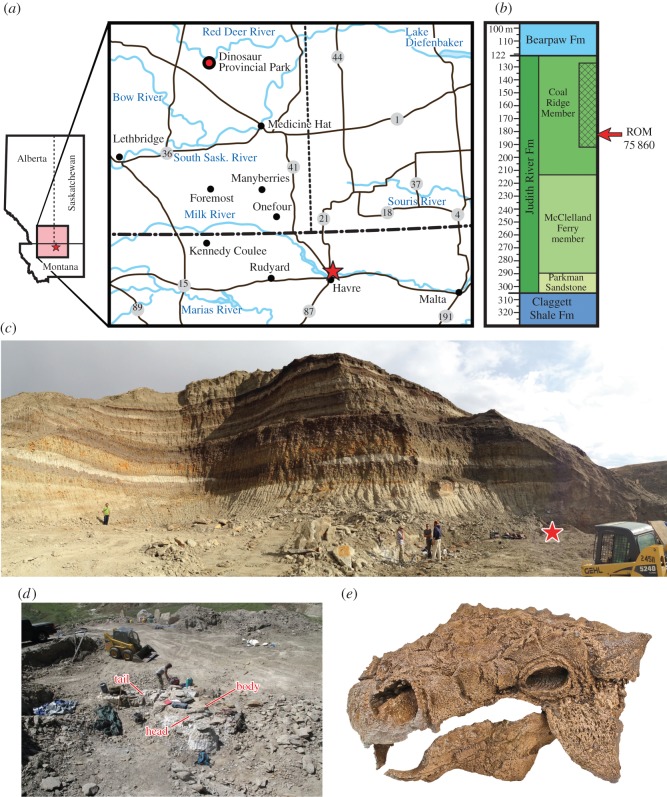
Holotype locality for ROM 75860, *Zuul crurivastator*. (*a*) Map showing the geographical location of ROM 75860, marked with a star, near Havre, Montana. (*b*) Approximate stratigraphic range of the host stratum of ROM 75860 within the Coal Ridge Member of the Judith River Formation. (*c*) Panoramic view of the *Z. crurivastator* quarry, with the location of the specimen marked by a star. (*d*) Orientation of ROM 75860 in the quarry during excavation. (*e*) Well-preserved skull and jaws of ROM 75860 showing quality of preservation.

ROM 75860 was discovered and excavated by a private commercial fossil company in 2014 (figure 1). The skeleton was excavated primarily as two large blocks, one containing the skull and torso, and one containing the tail. The skull and lower jaws were subsequently prepared out of the large body block, and the tail block was partially prepared (in dorsal view) prior to acquisition by the Royal Ontario Museum. The holotype quarry was visited by DCE on 20 August 2014 and 16–20 July 2016 in order to confirm the stratigraphic position and geological context of the specimen. Site photographs and notes were taken during both of these visits, and more extensive geological investigations were undertaken in 2016. A measured geological section, along with detailed palaeoenvironmental and taphonomic analyses, will be presented in a subsequent paper.

**Figure 2.**
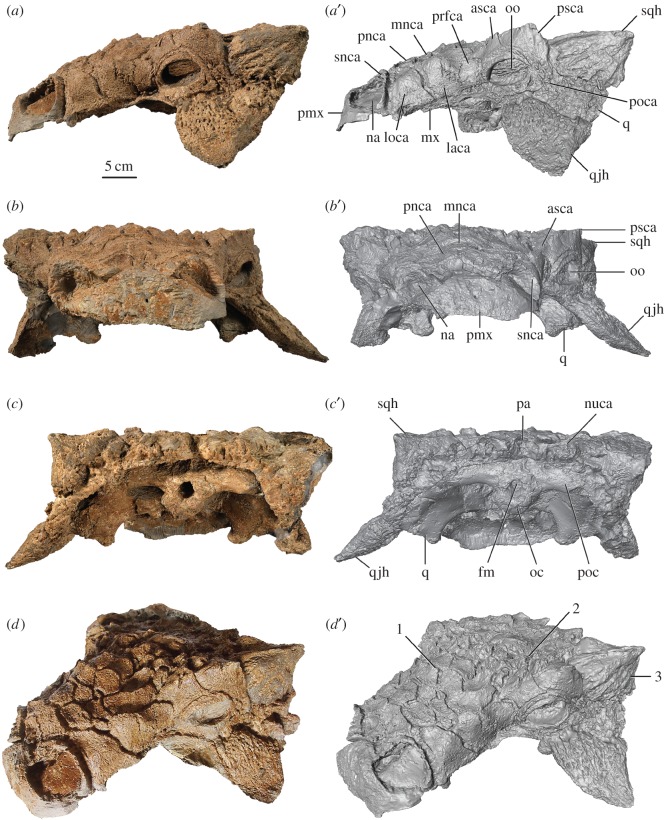
Holotype of *Zuul crurivastator*, ROM 75860, skull in (*a*) left lateral, (*b*) anterior, (*c*) posterior and (*d*) oblique left anterodorsal views. (*a*′–*d*′) are digital models of the skull. Diagnostic characters for *Z. crurivastator* include (1) imbricated frontonasal caputegulae, (2) peaked, pyramidal prefrontal and middle supraorbital caputegulae and (3) prominent apicobasal furrows on the squamosal horns. Abbreviations are as follows: asca, anterior supraorbital caputegulum; fm, foramen magnum; laca, lacrimal caputegulum; loca, loreal caputegulum; mnca, median nasal caputegulum; mx, maxilla; na, external naris; nuca, nuchal caputegulum; oo, ocular osteoderm; oc, occipital condyle; pa, parietal; pmx, premaxilla; pnca, postnarial caputegulum; poc, paroccipital process; poca, postocular caputegulum; prfca, prefrontal caputegulum; psca, posterior supraorbital caputegulum; q, quadrate; qjh, quadratojugal horn; snca, supranarial caputegulum; sqh, squamosal horn.

**Figure 3.**
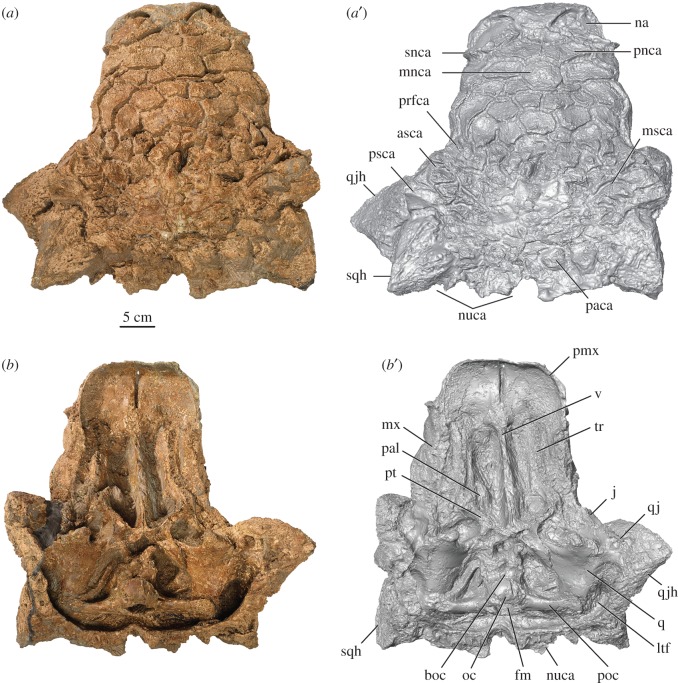
Holotype of *Zuul crurivastator*, ROM 75860, skull in (*a*) dorsal and (*b*) ventral views. (*a*′,*b*′) are digital models of the skull. Abbreviations are as follows: asca, anterior supraorbital caputegulum; boc, basioccipital; fm, foramen magnum; j, jugal; ltf, laterotemporal fenestra; mnca, median nasal caputegulum; msca, middle supraorbital caputegulae; mx, maxilla; na, external naris; nuca, nuchal caputegulum; oc, occipital condyle; paca, parietal caputegulum; pal, palatine; pmx, premaxilla; pnca, postnarial caputegulum; poc, paroccipital process; prfca, prefrontal caputegulum; psca, posterior supraorbital caputegulum; pt, pterygoid; q, quadrate; qj, quadratojugal; qjh, quadratojugal horn; snca, supranarial caputegulum; sqh, squamosal horn; tr, tooth row; v, vomer.

A three-dimensional model of the cranium of ROM 75860 was created using an Artec Spyder handheld laser scanner, and the acquired point cloud data were processed in the software program Artec Studio 11. Finally, the resultant stereolithography file (.STL) was manipulated in Avizo 6.1.1; the surface rendering was set to vertex normal, view set to orthographic, and the colour and lighting was adjusted to produce the reconstructions presented in figures 2 and 3. Morphological variation among ankylosaurids was assessed through direct observation of specimens or published literature (electronic supplementary material, S1) and measurements. All measurements were taken using digital calipers or measuring tape. Nomenclature for ankylosaurid cranial ornamentation follows Arbour & Currie [16]; we use the term ‘caputegulum’, coined by Blows [17], to describe the ornamentation on the dorsal surface of ankylosaurid skulls.

We assessed the phylogenetic position of *Zuul* using the character-taxon matrix for Ankylosauridae presented in Arbour *et al*. [18], which includes 58 taxa and 177 characters (electronic supplementary material, S1 and S2), and compiled in Mesquite v. 3.04 build 725 [19]. We follow all taxonomic opinions presented in [20] (but see also [21] for an alternative taxonomic hypothesis for Mongolian ankylosaurines); in particular we consider *Anodontosaurus lambei*, *Dyoplosaurus acutosquameus* and *Scolosaurus cutleri* distinct from *Euoplocephalus tutus*, and consider *Oohkotokia horneri* a junior synonym of *S. cutleri* [16]. We performed a cladistic parsimony analysis using the Traditional Search option in TNT v. 1.5 [22–24]; all characters were treated as unordered and of equal weight, and we used the tree-bisection reconnection (TBR) swapping algorithm with 1000 replications. We calculated Bremer support values using TBR of all existing trees, and bootstrap values using standard sampling with replacement and 1000 replicates.

### 2.1. Nomenclatural acts

The electronic edition of this article and the nomenclatural acts it contains have been registered in ZooBank, the online registration system for the International Code of Zoological Nomenclature, and fulfils the requirements of the amended ICZN. The ZooBank LSID (Life Science Identifiers) for this publication is: URN:LSID:ZOOBANK.ORG:ACT:5D8D582F-718C-4416-B12C-A31CA2701779. The electronic edition of this work was published in a journal with an ISSN, and has been archived and is available from the following digital repositories: PubMed Central, CLOCKSS and PORTICO. The new names published here are available under the ICZN from the electronic edition of this article.

## 3. Occurrence and geological setting

ROM 75860 was collected on the northern outskirts of the city of Havre, Montana, from badlands exposed on the north side of the Milk River, in Section 35, Township 33N, Range 15E. The specimen occurred in a multi-taxic bonebed hosted at the base of an approximately 3 m thick sandstone channel complex within a mudstone-dominated alluvial succession. The quarry of ROM 75860 occurs within strata that are part of the Coal Ridge Member of the Judith River Formation [1], representing a coastal plain alluvial succession deposited during transgression of the Bearpaw Sea [1,25,26]. Eberth & Hamblin [27] published a measured section based on the outcrop from which the quarry site is located. The thickness of the strata in the vicinity of the quarry is over 45 m, since the outcrop extends below the measured section presented in Eberth & Hamblin [27] (D Eberth 2016, personal communication). Eberth & Hamblin [27] interpreted these strata to represent the top of the Oldman Formation and the southeastern extent of the Dinosaur Park Formation in northern Montana. More recently, these strata have been interpreted as belonging to the upper Judith River Formation by Rogers *et al*. [1]. The total thickness of the Judith River section in the Havre area, taken from a well log north of the city [1, fig. 12], is approximately 180 m. The mid-Judith discontinuity (MJD), which marks the boundary between the lower McClelland Ferry Member and the upper Coal Ridge Member of the Judith River Formation, and the regressive and transgressive expressions of the formation, respectively, is identified in the middle of the 180 m thick unit in subsurface log [1, fig. 12], and is below the outcrop in the Havre area. The host stratum of ROM 75860 occurs 12 m from the top of the quarry section, and approximately 10 m below the base of the measured section in Eberth & Hamblin [27]. This places the quarry approximately 55 m from the top of the stratigraphically highest beds of the Judith River Formation in the Havre area, which does not include the Bearpaw Shale but shows coal development within the top 20 m of section [26, fig. 15], suggesting a close stratigraphic proximity to the upper contact of the Judith River Formation (probably within 10 m, based on Eberth & Hamblin [27]). This, therefore, places the quarry within the middle part of the Coal Ridge Member of the Judith River Formation. Based on the reported age estimates for deposition of this member, the host stratum of ROM 75860 is, therefore, between 76.2 and 75.2 Myr old.

ROM 75860 was discovered accidentally on 16 May 2014 during overburden removal for a scattered tyrannosaurid skeleton, when a skid-steer loader encountered the tail club knob. It was found approximately 10 m from the edge of the exposure, and beneath more than 12 m of overburden. As such, none of ROM 75860 had been subject to modern surface erosion prior to its discovery, and it is in an exceptional state of preservation. Most of the skeleton was preserved in a cemented sandstone concretion within the base of a channel. The tail, pelvis and dorsal vertebrae are articulated, with some of the dorsal ribs slightly displaced. The skull, lower jaws and cervical vertebrae were disarticulated but very closely associated with the anterior end of the skeleton. The skeleton was preserved upside-down with the ventral surface facing upwards. It was excavated as two large blocks, one containing the skull and torso, and the other containing the tail. The skull and lower jaws (along with a few other associated postcranial bones and osteoderms) have been completely removed from the surrounding rock. The torso block, weighing more than 15 000 kg, is currently undergoing preparation. The quarry also produced the remains of numerous other taxa, including turtles, crocodilyforms, theropods, hadrosaurids, invertebrates and plants. Like ROM 75860, these fossils also represent some of the best preserved and most complete examples of their respective taxa from the Judith River Formation and will be described in future publications.

## 4. Systematic palaeontology

Dinosauria Owen, 1842 [28]

Ornithischia Seeley, 1887 [29]

Thyreophora Nopcsa, 1915 [30]

Ankylosauria Osborn, 1923 [31]

Ankylosauridae Brown, 1908 [32]

Ankylosaurinae Brown, 1908 [32]

Ankylosaurini Arbour and Currie, 2016 [20]

*ZUUL* gen. nov.

*Type and only known species*: *Zuul crurivastator* gen. et sp. nov.

*Etymology:* The generic name refers to Zuul the Gatekeeper of Gozer, a fictional monster from the 1984 film *Ghostbusters*, and the species epithet combines crus (Latin) for shin or shank, and vastator (Latin) for destroyer, in reference to the sledgehammer-like tail club.

**Figure 4.**
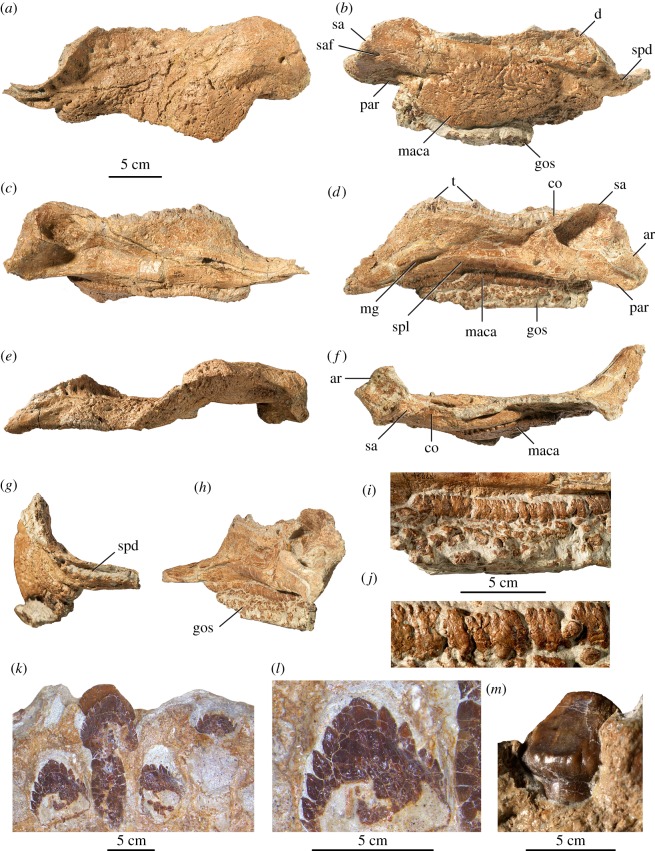
Holotype of *Zuul crurivastator*, ROM 75860, lower jaws and dentition. (*a*) Left lower jaw in lateral view, (*b*) right lower jaw in lateral view, (*c*) left lower jaw in medial view, (*d*) right lower jaw in medial view, (*e*) left lower jaw in ventral view, (*f*) right lower jaw in dorsal view, (*g*) right lower jaw in anterior view, (*h*) right lower jaw in posterior view, (*i*) ossicles preserved on the ventral side of the right lower jaw, (*j*) detail view of the large square ossicles abutting the medial surface of the right lower jaw, (*k*) *in situ* teeth in the right lower jaw, lingual view, anterior is to the left, (*l*) detail view of the most anterior tooth in (*k*), lingual view and (*m*) highly worn *in situ* tooth in the right lower jaw, labial view. Abbreviations are as follows: ar, articular; co, coronoid; d, dentary; gos, gular ossicles; maca, mandibular caputegulum; mg, Meckelian groove; par, prearticular; sa, surangular; saf, surangular foramen; spd, sulcus for the predentary; spl, splenial; t, tooth.

**Figure 5.**
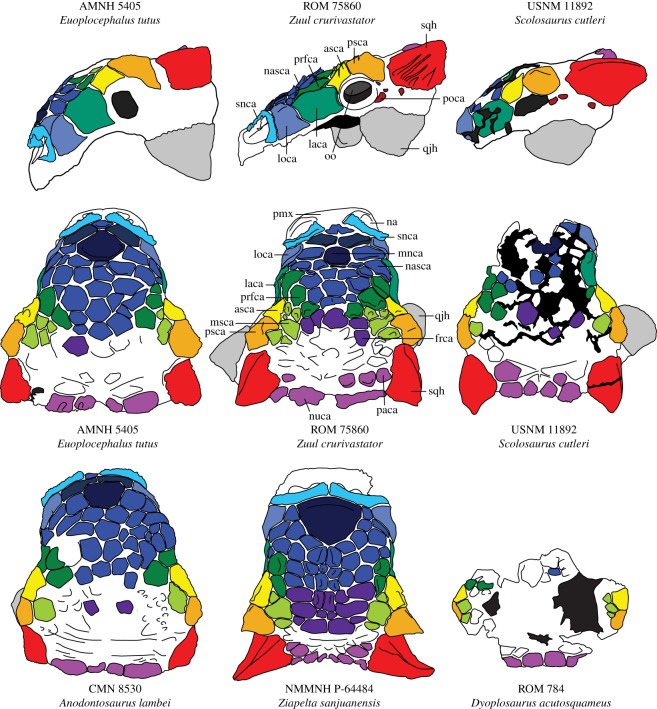
Cranial ornamentation of ankylosaurins compared, in dorsal and lateral views. Abbreviations are as follows: asca, anterior supraorbital caputegulum; frca, frontal caputegulum; laca, lacrimal caputegulum; loca, loreal caputegulum; mnca, median nasal caputegulum; msca, middle supraorbital caputegulum; na, external naris; nasca, nasal caputegulae; nuca, nuchal caputegulum; oo, ocular osteoderm; paca, parietal caputegulum; pmx, premaxilla; pnca, postnarial caputegulum; poca, postocular caputegulum; prfca, prefrontal caputegulum; psca, posterior supraorbital caputegulum; qjh, quadratojugal horn; snca, supranarial caputegulum; sqh, squamosal horn. Skulls are scaled to the same anteroposterior length.

**Figure 6.**
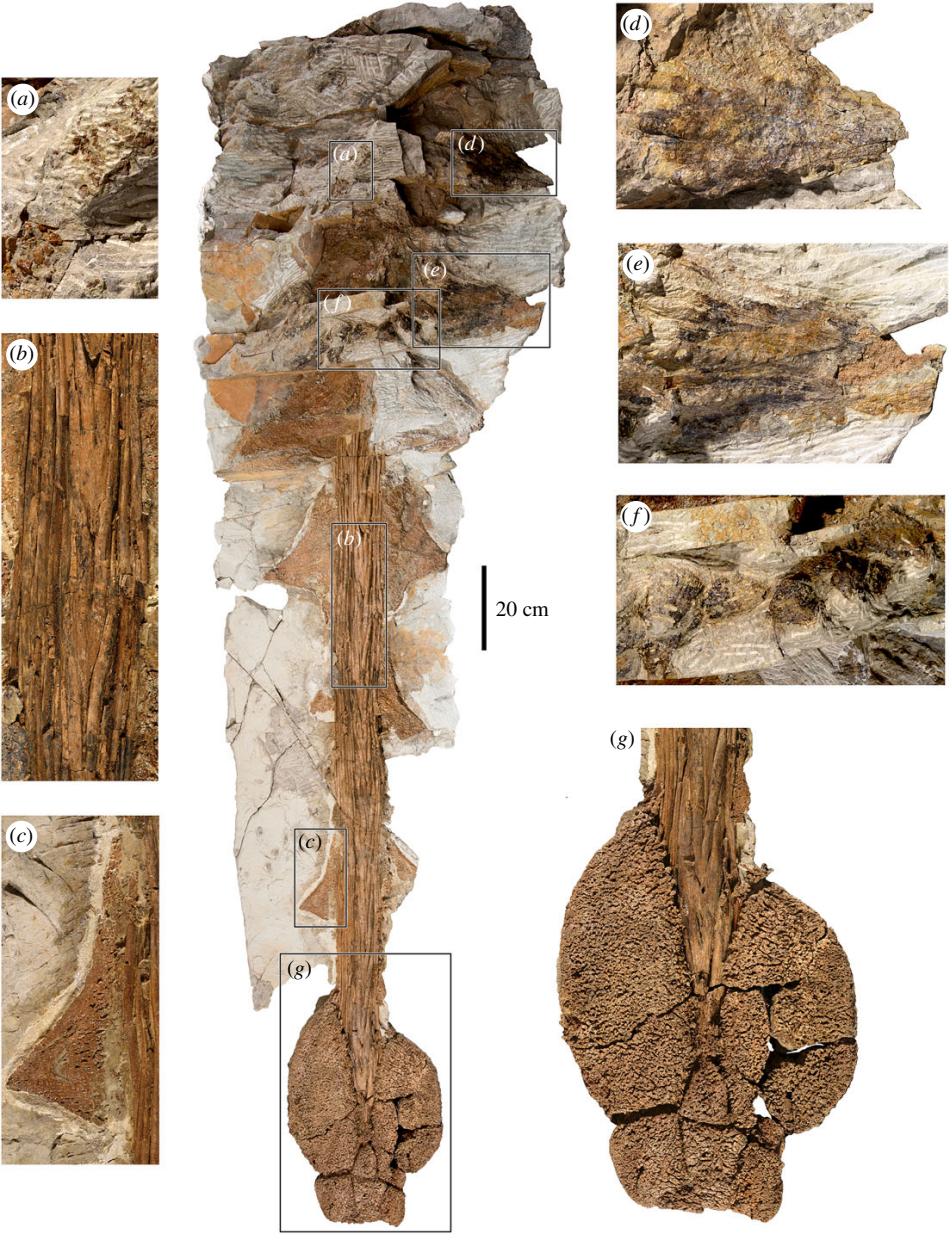
Overview of the tail of *Zuul crurivastator* ROM 75860 in dorsal view, with insets of detailed anatomy. (*a*) Field of ossicles in the anterior portion of the tail. (*b*) Detail of the neural arches of the handle caudal vertebrae, and ossified tendons. (*c*) Left caudal osteoderm from the seventh pair. (*d*) Preserved epidermal sheath on the right caudal osteoderm from the second pair. (*e*) Preserved epidermal sheath on the right caudal osteoderm from the third pair. (*f*) Epidermal scales lacking bony cores, arranged in a transverse row at the third pair of caudal osteoderms. (*g*) Close-up of the tail club knob.

**Figure 7.**
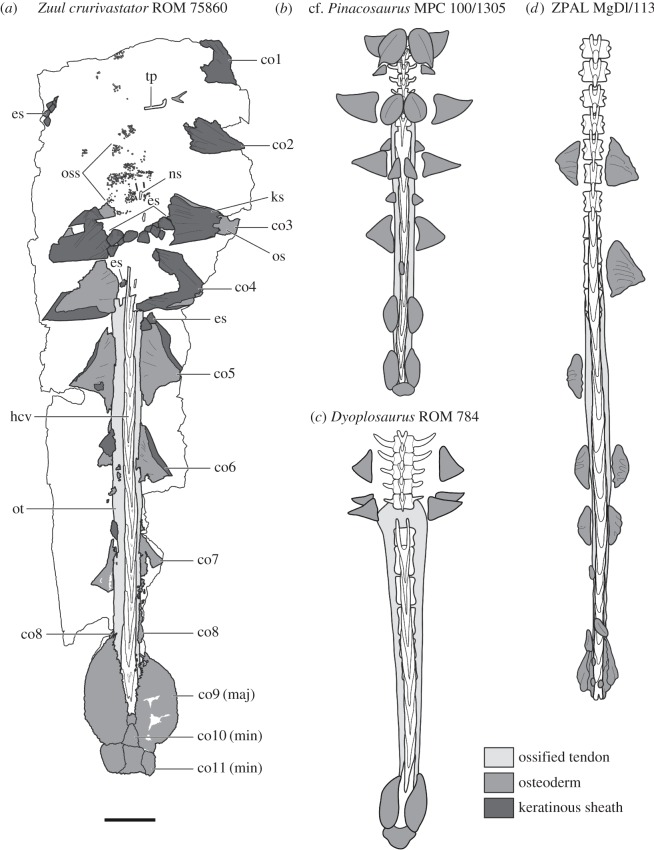
Interpretive illustration of the tail of *Zuul crurivastator* compared with other Mongolian and North American ankylosaurines, in dorsal view, showing differences in the morphology of the caudal osteoderms, and relationships between dermal and epidermal elements: (*a*) *Z. crurivastator*, ROM 75860, (*b*) cf. *Pinacosaurus*, MPC 100/1305, (*c*) *Dyoplosaurus acutosquameus*, ROM 784, (*d*) Ankylosaurinae indet. from the Nemegt Formation, ZPAL MgD I/113. Vertebrae are indicated in white, zones of ossified tendons are indicated by the lightest grey, osteoderms and ossicles are indicated by a medium grey, and epidermal osteoderm sheaths and scales are indicated by the darkest grey. Abbreviations are as follows: co, caudal osteoderm; co (maj), major osteoderm of the tail club knob; co (min), minor osteoderm of the tail club knob; es, epidermal scale; hcv, handle caudal vertebra; ks, keratinous sheath; ns, neural spine; oss, ossicle; ot, ossified tendons; tp, transverse process. Scale bar, 20 cm.

*Holotype:* ROM 75860, figures 1–7.

*Holotype locality:* Approximately 5 km northwest of the John Wodarz Bridge, in badlands exposures of the Judith River Formation outcropping along the north side of the Milk River Drainage near the City of Havre, Montana (Section 35, Township 33N, Range 15E). GPS coordinates for the quarry are reposited at the Royal Ontario Museum. The site was originally designated Theropoda Exp LLC Locality LC02(Q).

*Stratigraphic horizon and age:* Coal Ridge Member of the Judith River Formation. The Coal Ridge Member was deposited between 76.2 and 75.2 Ma [1].

*Diagnosis:* Differs from all ankylosaurids in the possession of the following autapomorphies: imbricated, peaked frontonasal and frontoparietal caputegulae; prominent longitudinal furrows on the lateral surface of the squamosal horn; lateral caudal osteoderms along the tail club (excluding the knob osteoderms) are strongly concave on the leading edge with posteriorly offset apices; tail club knob dorsoventrally flat, with height to length ratio less than 0.20. *Zuul* can be further differentiated from other ankylosaurins in the following traits: possesses pyramidal prefrontal, frontoparietal and middle supraorbital caputegulae (unlike the conical caputegulae in *Nodocephalosaurus* and *Talarurus*); squamosal horns extend posteriorly well past the nuchal shelf, similar to *Scolosaurus* but unlike *Anodontosaurus*, *Euoplocephalus*, or *Ziapelta*; postocular caputegulae small and sparsely distributed, similar to *Scolosaurus* but unlike *Anodontosaurus*, *Euoplocephalus* or *Ziapelta*. Caudal osteoderms lateral to tail club handle are proportionately larger and more sharply pointed than in ankylosaurines from the Nemegt Formation of Mongolia.

## 5. Osteological description

ROM 75860 is a partial skeleton consisting of a nearly complete cranium, both lower jaws, and a partially articulated postcranium (figures 1–7). At present, the skull and lower jaws are fully prepared (figures 2–4) and a block containing free caudal vertebrae and the complete tail club is partially prepared (figures 6 and 7). The main body block weighs over 15 000 kg, and preparation will take several years to complete. Currently exposed on this block are disarticulated cervical vertebrae, articulated dorsal and sacral vertebrae, dorsal ribs, pelvic elements, osteoderms, small ossicles and skin impressions. The specimen preserves numerous features of the integument, including osteoderms (both disarticulated and *in situ*), millimetre-sized ossicles, and skin impressions. Disarticulated but associated osteoderms are preserved in the block containing the torso, and ossicles are preserved on the ventral surface of the left lower jaw, around the torso, and on the tail. Large triangular osteoderms in the anterior region of the tail are covered in a shiny black material that may represent the original keratinous sheath. Biochemical analyses are required to confirm the presence of keratin and other preserved soft tissues in ROM 75860. Here we focus on the description of the skull and tail, which are among the most taxonomically informative elements for ankylosaurines.

## 6. Skull

The skull is complete, missing only the tip of the right quadratojugal horn and the ventral edge of the vomers (figures 2, 3 and 5). The skull has undergone plastic deformation, with some dorsoventral compaction and mediolateral skewing evidenced by the bilateral asymmetry of the skull overall, and flattened, oval orbits [33]. The lateral sides of the snout taper gently towards a squared-off premaxillary beak. Laterally, the skull is relatively flat dorsally, unlike the arched profile of some specimens of *Euoplocephalus*, *Anodontosaurus*, *Scolosaurus* and *Ziapelta*, but this might result from taphonomic compaction of the skull. As in other ankylosaurines, the antorbital fenestra is absent and the supratemporal fenestrae are obscured. In dorsal view, the skull has a trapezoidal outline. Apart from the three known skulls of *Ankylosaurus* (AMNH 5214, AMNH 5895 and CMN 8880), ROM 75860 is the largest ankylosaurine skull recovered from Laramidia (table 1).

**Table 1.** Dimensions of the skull of *Zuul crurivastator* (in millimetres) compared with other ankylosaurins. (R) refers to the right side.

specimen	width across supraorbitals	width across squamosal horns	width immediately anterior to orbits	width across loreal caputegulae	premaxillary-occipital condyle length (ventral)	premaxillary-nuchal shelf length (dorsal)	length of lower jaw
	*Ankylosaurus magniventris*						
AMNH 5895	525						
AMNH 5214	490	640			542		
	*Zuul crurivastator*						
ROM 75860	393	415	265	232	395	501	309 (R)
	*Euoplocephalus tutus*						
ROM 1930	388	392	301		368		
TMP 1979.14.164	385	399					
AMNH 5405	372	385	225		384		292
AMNH 5337	365				392		310
AMNH 5403	353	365	252		410		318
TMP 1996.127.1	320	303	240		370		
UALVP 31	313	278		242		355	259
CMN 0210				201			
	*Anodontosaurus lambei*						
TMP 1997.132.1	355	357			378		
AMNH 5238	341	339	258		345	363	
CMN 8530	319	300	195	212	352		
ROM 832			280				
AMNH 5223			230				
	*Dyoplosaurus acutosquameus*						
ROM 784	355						
	*Scolosaurus cutleri*						
MOR 433	354	383	312				
USNM 11892	322	317	210				
TMP 2001.42.1	263	288					
	*Ziapelta sanjuanensis*						
NMMNH P64484	320	390				360	

### 6.1. Rostral region

The premaxillae form a broad, square, edentulous beak (figures 2 and 3). Ventral to the external nares the premaxillae are smooth. The contact between the premaxilla and maxilla is obscured by cranial ornamentation; in juvenile *Pinacosaurus* (e.g. ZPAL MgD II/1) where the sutures are visible, the contact lies behind the supranarial ornamentation and is marked by a change in angle between the premaxillary and maxillary tomium.

The external nares are broad, anteriorly oriented ovals (figures 2 and 3). Distinct paranasal apertures are not visible within the nasal vestibule, and a single narial aperture (aperture A *sensu* [34]) is located medially and posteriorly within the vestibule. The external nares are rimmed dorsally by the arched supranarial caputegulae, which are rugose and anteroventrally narrow, with laterally offset apices.

The frontonasal caputegulae form a continuous mosaic of low-relief tiles over the frontals and nasals, obscuring their contacts with each other and with other cranial bones (figure 3). These caputegulae are imbricated and have upraised posterior edges and peaks; the imbrication becomes stronger, and the caputegulae more peaked, towards the frontal region compared with the nasal region. Posterior to the supranarial caputegulae are a small medial pair of caputegulae and a larger, semi-rectangular pair of postnarial caputegulae. The postnarial caputegulae bracket the anterior edge of the median nasal caputegulum, a prominent and distinctive hexagonal caputegulum located on the midline of the skull. An additional pair of semi-rectangular caputegulae flank the lateral edges of the median nasal caputegulum. A second hexagonal midline caputegulum is located anterior to the supraorbitals. Posterior to the median nasal caputegulum, the frontonasal caputegulae take on approximately hexagonal or square outlines.

The maxillae are located posterior to the premaxillae and are mostly covered by the loreal caputegulae (figure 2). The approximately rectangular loreal caputegulum is located immediately behind the supranarial caputegulum on the lateral surface of the rostrum. It extends onto the dorsal surface of the rostrum and is taller than wide. Ventrally the maxilla is exposed without ornamentation, and forms a sharp, medially inset tomial ridge.

The lacrimal is entirely obscured by the lacrimal caputegulum and its boundaries with other bones are not visible (figure 2); in juvenile *Pinacosaurus* (e.g. ZPAL MgD II/1), the lacrimal contributes to the anterior edge of the orbit and is a blocky, rectangular bone. Like the loreal caputegulum, the lacrimal caputegulum is also rectangular, but is wider than tall, and is located immediately posterior to the loreal caputegulum and anterior to the orbit. Only a thin edge of the lacrimal caputegulum is visible in dorsal view.

### 6.2. Temporal region

The boundaries between the frontal and parietal bones are obscured by the frontoparietal ornamentation (figure 3). Caputegulae in the presumed frontoparietal region are slightly less distinct than those in the frontonasal region, and there is a smooth, striated central region of the parietal or posterior frontals that is devoid of caputegulae. An abrupt, deep depression mars the midline caputegulum in the posterior frontoparietal region, and a smaller depression is located medial to the right posterior supraorbital. These depressions do not have any reactive new bone growth, and it appears as though the caputegulae dive down into the depressions; as such, these depressions probably represent taphonomic artefacts rather than pathologies or foramina. The parietal overhangs the posterior region of the skull, forming a nuchal shelf that obscures the braincase in dorsal view. Four nuchal caputegulae are present at the posterior margin of the skull, and four upraised parietal caputegulae imbricate over the nuchal caputegulae. The parietal is fused to the supraoccipital and paroccipital processes.

Two distinctive supraorbital caputegulae roof the lateral edges of the orbital cavity (figure 3). The anterior supraorbital caputegulum is smaller than the posterior caputegulum. Both are sharply pointed, but together form a continuous edge along their lateral sides. The supraorbitals are prominent and project strongly laterally. Shallow, indistinct, transversely oriented furrows are present on the dorsal surface of each supraorbital caputegulum. A raised rim of ornamentation lies below the supraorbital caputegulae and above the orbit in lateral view. A third supraorbital bone is visible in juvenile *Pinacosaurus* (ZPAL MgD II/1) medial to the anterior and posterior supraorbitals, and contributes to the roof of the orbital cavity but not the lateral edge of the orbit. This region in ROM 75860 does not bear a single distinct caputegulum as for the anterior and posterior supraorbital bones, but instead carries multiple small pyramidal caputegulae with small bases and tall peaks.

The boundaries of the postorbital cannot be distinguished in ROM 75860, but in juvenile *Pinacosaurus* (ZPAL MgD II/1) this bone is located posterior to the posterior supraorbital, forms part of the posterolateral corner of the skull (potentially contributing to the base of the squamosal horn), and contributes to the postocular shelf.

The squamosal horns form the dorsal posterolateral corner of the skull and are robust and pyramid-shaped (figures 2 and 3). They are relatively long and backswept, with apices that project well past the nuchal crest. Shallow but distinct furrows are oriented longitudinally along the lateral surfaces of the squamosal horns and converge towards the apex. There is a sharp keel on the dorsal surface of each horn.

The boundary between the lacrimal and jugal is difficult to discern, but the jugal probably forms the bulk of the ventral border of the orbit and the floor of the orbital cavity, based on its morphology in juvenile *Pinacosaurus* (ZPAL MgD II/1). The jugal bears irregular, rugose ornamentation (figure 2). The orbits are oval rather than circular, indicating that some dorsoventral compaction has occurred [33]. An osteoderm is preserved within each orbit (figure 2). These free-floating osteoderms were originally located within the eyelid and are known in the holotype of *Dyoplosaurus* and several specimens of *Euoplocephalus* [16,35]. The lateral surfaces of these osteoderms are highly rugose, with a slightly striated appearance.

Irregular postocular caputegulae are present behind the left orbit on the squamosal and quadratojugal (figure 2). The largest postocular caputegulum is located at the posteroventral corner of the orbit, and several smaller and more irregularly shaped caputegulae are located along the posterior margin of the orbit and ventral margin of the squamosal horn.

The quadratojugal horns form the ventral posterolateral corners of the skull (figures 2 and 3). The right quadratojugal horn of ROM 75860 is incomplete but the left quadratojugal horn is complete. The broken edge of the right quadratojugal horn reveals that the horn is most likely both an outgrowth of the quadratojugal itself, and enveloped by a co-ossified osteoderm. The quadratojugal horns are massive and triangular in lateral view, with a sharp, posteriorly offset apex. The anterior edge is gently curved, and the posterior edge is straight. Shallow, irregular, radiating furrows are present on the external sides of the horns. The quadratojugal horn contacts the jugal, and obscures the quadrate in lateral view.

### 6.3. Palatal region

The ventral surfaces of the premaxillae form part of the secondary palate (figure 3*b*). A sagittal slit divides the gently concave premaxillary palate in half. Posteriorly, the premaxillae contact the vomer at the midline, and the maxillae more laterally. The contact with the maxilla is marked by an indistinct transverse suture.

The tooth rows are inset from the lateral side of the maxillae, creating a buccal emargination (figure 3). The tooth rows converge medially and diverge anteriorly and posteriorly. Several teeth are preserved in the alveoli, and approximately 18–20 alveoli are preserved in each row along a distance of about 13 cm. Matrix obscures the posterior portion of the maxillae and their contribution to the internal choanae.

The paired vomers are narrow, sheet-like bones, approximately 18 cm long and closely appressed to each other (figure 3). The ventral edges of the vomers are broken, but approach the same level as the tooth rows, dividing the oral cavity into two longitudinal partitions. Matrix obscures the palatines and their relationship to the vomers.

The paired pterygoids form a W-shaped structure posterior to the secondary palate and anterior to the braincase (figure 3). The pterygoid body is transversely and slightly anteroposteriorly oriented, and contacts the basipterygoid process. No pterygoid foramina are present. The pterygoid flange projects anterolaterally, tapering distally into a blunt terminus. The quadrate ramus projects posterolaterally and contacts the quadrate with a scarf joint. Ectopterygoids cannot be distinguished in ROM 75860.

### 6.4. Occipital/Basicranial Region

The basisphenoid is an unpaired element located posterior to the pterygoids and anterior to the basioccipital (figure 3). Anteriorly, it is bifurcated into elongate and well-separated basipterygoid processes, which contact the posterior surface of the pterygoids. A transverse rugose ridge on the ventral surface of the braincase represents the basal tubera and the contact between the basisphenoid and the basioccipital. The posterior floor of the braincase and the occipital condyle are formed by the basioccipital. The occipital condyle is reniform and oriented posteroventrally, and is not offset from the rest of the basioccipital by a constricted neck. It is obscured in dorsal view by the nuchal crest (figure 3).

The supraoccipital is an unpaired median bone that is fused to the ventral surface of the parietals and forms the dorsal border of the foramen magnum (figure 2). The paroccipital processes are formed via the fusion of the exoccipitals and opisthotics. The paroccipital process extends laterally from the foramen magnum, and contacts the quadrate laterally and fuses with the parietal dorsally. The lateral terminus is only moderately dorsoventrally expanded. They are obscured by the nuchal shelf in dorsal view.

The anteroventrally inclined quadrate meets the pterygoid anteriorly, the quadratojugal laterally and the exoccipital posteriorly (figure 3). The quadrate is co-ossified to the quadratojugal but not to the pterygoid or exoccipital. The quadrate condyles are bean-shaped in ventral view, with a greater anteroposterior length medially than laterally. The ventral surface of the condyle is weakly saddle-shaped.

### 6.5. Lower jaws

ROM 75860 includes both complete lower jaws, but a predentary has not yet been identified (figure 4). The lower jaw is long, low and lacks a mandibular fenestra. The lateral surface is partially covered by the roughly triangular mandibular caputegulum, completely obscuring the angular and obscuring some of the boundaries of the surangular and dentary. This ornamentation probably represents sculpturing and outgrowth of the bones themselves, rather than co-ossification of an osteoderm, based on the presence of light sculpturing on the angular in juvenile *Pinacosaurus* (ZPAL MgD II/1). The retroarticular process is short and broad, and the quadrate articular fossa is medially offset from the tooth row. The adductor fossa is relatively small and shallow, representing only about 20% the length of the lower jaw.

The dorsal margin of the dentary is sinusoidal and the alveolar border is medially convex (figure 4). Anteriorly, the dentary abruptly tapers into a dorsoventrally shortened ramus that curves medially at approximately 90°. The anterior edge of this ramus has a well-defined sulcus for the predentary. The alveoli on both dentaries are slightly obscured by matrix, but the right dentary preserves 28 alveoli. The tooth row on the right dentary measures 160 mm in length.

The splenial is visible on the medial and ventral surfaces of the lower jaw (figure 4). Its contact with the dentary is marked by a prominent anteriorly located, horizontally oriented aperture and a distinctive longitudinal groove (the Meckelian groove). Posteriorly, the splenial extends underneath the prearticular. The mandibular caputegulum extends below the ventral margin of the splenial.

The surangular forms most of the posterodorsal region of the lower jaw in lateral view and contributes to the retroarticular process (figure 4). A faint vertically oriented suture demarcates the boundary between the surangular and the dentary in lateral view. The boundary between the surangular and coronoid is indistinguishable, and together these form a continuous dorsal border with the dentary. The coronoid contributes to the anterior wall and the surangular contributes to the lateral wall of the adductor fossa.

The prearticular, with the surangular, forms the retroarticular process of the lower jaw (figure 4). The prearticular has a bluntly rounded posterior margin. Anteriorly it forms the shallow medial wall of the adductor fossa. It is overlain by the articular, which forms the quadrate articular fossa. The articular is mediolaterally wide and gently concave.

Numerous osteoderms less than a centimetre in diameter (hereafter referred to as ossicles) are preserved along the ventral margin of the right lower jaw (figure 4*i,j*). The ossicles closest to the jaw overlap the mandibular caputegulum medially. These are the largest gular ossicles, at 6–14 mm in width and 12–14 mm in height; two of these have co-ossified, with a total width of 24 mm. They are square to rectangular with distinct corners, and are aligned in a row abutting the mandibular caputegulum and splenial. Ventral to this row of gular ossicles, the ossicles become smaller (diameter 6 mm or less), more hexagonal or diamond-shaped, and are arranged in rosettes.

### 6.6. Dentition

The teeth are leaf-shaped, labiolingually compressed, and have long, straight roots (figure 4*k–m*). The crowns preserved in the anterior end of the right dentary are about 6.1 mm wide along the base, and about 7 mm tall. The crowns in this part of the tooth row bear 12–14 diamond-shaped cusps, and the apex cusp is slightly posteriorly offset. A rounded cingulum is present along the base of the crown. A highly worn tooth visible in lateral view on the left dentary has a deep wear facet that occupies the entire lateral surface of the crown, and no cusps remain (figure 4*m*).

## 7. Tail

A complete articulated tail is preserved in ROM 75860 (figures 6 and 7); the block containing most of the tail (except for a few anterior caudals or sacrocaudals, potentially) is 287 cm long. The free caudal vertebrae in the anterior half of the tail are only minimally exposed at present because skin impressions and soft tissues occur in this region; one transverse process and two neural spines are visible. A complete tail club is exposed in dorsal view, with ossified tendons and osteoderms preserved *in situ*. We follow the terminology of Coombs [36] in describing features of the tail club; a tail club includes both the cluster of enlarged, modified osteoderms at the tail tip (the knob) and the modified, interlocking distal caudal vertebrae (the handle). Thirteen caudal vertebrae are visible in the handle, and one additional vertebra may be obscured by matrix and soft tissues at the anterior end; based on CT scans of other ankylosaur tail club knobs [37], an additional 1–3 vertebrae might be obscured within the knob osteoderms. The tail club (including the 13 caudal vertebrae in the handle and the knob) is at least 210 cm long, making this the longest known tail club of any ankylosaurine from North America. Numerous integumentary structures are preserved on the tail, including *in situ* osteoderms, ossicles, the horny sheaths surrounding the osteoderms, and scale impressions.

Like other ankylosaurines, the vertebrae in the tail club handle are highly modified relative to the anterior free caudal vertebrae, and interlock tightly with each other to form a rigid, inflexible structure (figures 6 and 7). The prezygapophyses are highly elongated and extend more than 50% the length of the adjacent caudal centrum, and the articular faces are vertically oriented to embrace the neural spine of the adjacent vertebra. The neural spine is horizontally oriented with a flat dorsal surface, forming a wedge-shaped projection that is fully enveloped by the prezygapophyses of the adjacent caudal vertebra. In dorsal view, the prezygapophyses and neural spines form a series of interlocking V-shaped structures, and the average angle of divergence of the prezygapophyses is less than 20°. No centra are visible because they are obscured by ossified tendons, osteoderms and matrix. The tail club is approximately 210 cm long, with the anterior edge obscured by matrix and soft tissues at present. At the anterior edge of the knob, the handle vertebrae are 5.5 cm in width.

Bundles of long ossified tendons are closely appressed to the lateral sides of the handle, and pass between the terminal handle vertebrae and the medial surface of the knob osteoderms (figure 6). Up to eight tendons are stacked together on each side of the handle. The tendons are arranged in parallel along their lengths, but imbricate at the anterior and posterior ends. The tips are tapered and flattened. Their boundaries are difficult to trace, but many of the tendons exceed 50 cm in length and are 5–8 mm in width.

Osteoderms are preserved *in situ* along the entirety of the tail (figures 6 and 7). Of particular note in ROM 75860 is that these osteoderms are preserved not only in the anterior, flexible portion of the tail (as is known in other Laramidian ankylosaurin specimens, like ROM 784 and ROM 1930), but also along the tail club handle. This is the first occurrence of these osteoderms in a North American ankylosaurine. Five pairs of osteoderms (excluding the knob osteoderms) are preserved along the tail club handle, and three pairs were present anterior to the tail club. Using the standardized terminology for osteoderm position in a transverse row proposed by Burns & Currie [38], these osteoderms may be present in either the lateral or distal position, and no medial osteoderms are present, except on the tail club knob. Small irregular ossicles, similar to those preserved in the gular region, are interspersed among the larger osteoderms along the entire length of the tail.

The lateral caudal osteoderms are triangular, sharply pointed in dorsal view, and dorsoventrally compressed (figures 6 and 7, table 2). Anteriorly, the osteoderms have high aspect ratios with greater apicobasal than anteroposterior lengths, and straight anterior and posterior edges. These osteoderms are nearly equilateral, with only slightly posteriorly offset apices. Posteriorly, the apicobasal length decreases and the anteroposterior length increases. Starting at the fifth pair of osteoderms (the second pair on the tail club handle), the osteoderms have concave anterior edges that are much longer than the posterior edges, making the apex significantly offset posteriorly. The penultimate pair of lateral caudal osteoderms (pair number 8) before the knob are not triangular, but instead have rounded, irregular lateral edges. The left osteoderm in this pair appears to be overgrown by the left major knob osteoderm, with only a small sliver of the anterior edge visible.

**Table 2.** Measurements of the caudal osteoderms of ROM 75860, *Zuul crurivastator* (in millimetres). Osteoderm number positions correspond to those labelled in figure 7.

osteoderm position (anterior to posterior)	left or right side	anteroposterior length	apicobasal length	comments
1	R	—	—	only a portion of a horny sheath is visible at present
2	R	180	>250	encased in horny sheath, distal tip broken
3	R	190	>260	encased in horny sheath, distal tip broken
3	L	185		encased in horny sheath, distal tip broken, base of osteoderm visible
4	R	>210	>250	distal tip broken, covered by soft tissue medially which obscures anteroposterior length
4	L	>210	265	anterior edge obscured by soft tissues, distal tip slightly damaged
5	R	350	>170	distal tip broken, anteriormost edge obscured by soft tissue
5	L	365	184	
6	R	265	118	posterior edge damaged
6	L	—	—	badly damaged, missing posterior half and distal tip
7	R	∼200	87	medial and posterior edges broken
7	L	218	94	
8	R	110	21	dorsoventrally thin, rounded lateral edge
8	L	—	—	obscured by major knob osteoderm (left osteoderm 9)
9 (knob, major osteoderm)	R	352	165	semicircular in dorsal view
9 (knob, major osteoderm)	L	402	166	semicircular in dorsal view, overlaps left osteoderm 8
10 (knob, minor osteoderm)	midline	73	61 (greatest transversewidth)	triangular in dorsal view, with apex pointed anteriorly
11 (knob, minor osteoderm)	R	—	—	broken
11 (knob, minor osteoderm)	L	106	103	trapezoidal in dorsal view

The first three pairs of caudal osteoderms are covered with a black film, which appears to represent the preserved horny sheath that would have surrounded the osteoderm during life. The sheaths did not enlarge the overall spike morphology substantially, adding perhaps 1–2 cm to the outline of the osteoderm. The sheaths have distinctive apicobasal furrows and ridges. On the left osteoderm in the third pair, the base of the keratin sheath has a layered texture, similar to the texture observed at the base of bovid horn sheaths (e.g. *Bos* sp. ROM R293, *Capra hircus* ROM R972, *Redunca redunca* ROM R921). The osteoderms along the tail club handle do not preserve the horny sheaths over the entire surface, and reveal that the surface texture of the sheath does not reflect the surface texture of the underlying osteoderms. The caudal osteoderms are relatively smooth, with sparse pitting and shallow grooves.

The end of the tail includes a nearly complete tail club knob, the cluster of osteoderms enclosing the tail tip. Ankylosaurine tail club knobs are always composed of two major osteoderms, one on each side of the handle and which envelop the terminal caudal vertebrae laterally, dorsally and ventrally, plus a variable number of smaller minor osteoderms which cover the posterior tip of the tail [35]. The major osteoderms are often huge and comprise most of the mass of the tail club knob [16]. The knob of ROM 75860 measures 368 mm across its greatest width, 525 mm along its greatest length and 80 mm at its greatest height (figures 6 and 7, table 3). The left major osteoderm is anteroposteriorly longer than the right major osteoderm (but approximately the same apicobasal length), making the knob distinctively asymmetrical in dorsal view. The left major osteoderm has a small area of unusual ropy texture at the anterior edge, which overlaps the small lateral caudal osteoderm anteriorly. The major osteoderms meet closer to the midline dorsally than ventrally. They lack prominent keels or points on the lateral surfaces, and create an oval outline in dorsal view. The minor osteoderms are co-ossified and more difficult to discern, but in dorsal view there is a pair of trapezoidal osteoderms forming the posterior corners of the knob, one or more median osteoderms form the posterior edge of the knob, and a median triangular osteoderm encloses the dorsal surface of the knob. Together, the minor osteoderms give the posterior edge of the knob a squared-off appearance in dorsal view. The surface texture of the knob osteoderms is spongy, pitted and highly rugose, which differs from the more lightly pitted texture present on the other caudal osteoderms.

**Table 3.** Dimensions (in millimetres) of the tail club of *Zuul crurivastator* compared with other ankylosaurins. AMNH 5405 is broken in half with the middle portion containing the handle vertebrae missing; the left major osteoderm is 177 mm wide and the right is 181 mm wide. AMNH 5403 includes the complete width of the right major osteoderm and a portion of the left; the right major osteoderm is 179 mm wide. The proximal ends of the handle in TMP 2001.42.1, MACN Pv 12554 and ROM 788 are damaged and as such the original lengths may have been slightly longer. AMNH 5214 is mounted behind glass at an angle, and as such we estimated its width using ImageJ from figures in [39].

specimen	tail club length (handle + knob)	knob maximum width	knob maximum length	knob maximum height	width : length	height : length
	*Zuul crurivastator*					
ROM 75860	2100	368	525	80	0.70	0.15
	*Ankylosaurus magniventris*					
AMNH 5214		450	∼600		∼0.75	
	*Anodontosaurus lambei*					
AMNH 5211			300	173		0.58
AMNH 5245		595	316	220	1.88	0.70
TMP 1994.168.1		473	350	75	1.35	0.21
USNM 10753		425	398	210	1.07	0.53
	*Dyoplosaurus acutosquameus*					
ROM 784	1280	185	270	69	0.68	0.26
UALVP 47273	800	155	250	75	0.62	0.30
	*Euoplocephalus tutus*					
AMNH 5405		∼420	386	198	∼1.09	0.52
AMNH 5403		∼365				
	*Scolosaurus cutleri*					
TMP 2001.42.1	1550	316	291	190	1.08	0.65
	Dinosaur Park Formation, Ankylosaurinae indet.					
CMN 2234^a^			421	118		0.28
CMN 2251^a^			367	187		0.51
CMN 2252^a^			389	191		0.49
CMN 2253^a^			267	191		0.71
CMN 135		271	366	196	0.74	0.54
MACN Pv 12554	1670	470	400	170	1.17	0.43
ROM 788	1780	572	522	190	1.10	0.36
ROM 7761		133	>132	71		
TMP 1983.36.120		419	489	160	0.86	0.33
TMP 1993.36.421		360	375		0.96	
TMP 2000.57.03			240	186		0.77
UALVP 16247		355	415	100	0.86	0.24
	cf. *Pinacosaurus*					
PIN 614	1160		110			
	Djadokhta Formation, Ankylosaurinae indet.					
MPC 100/1305	1040	146	180	35	0.81	0.19
	Baruungoyot Formation, Ankylosaurinae indet.					
PIN 3142/251		220	246	57	0.89	0.23
	Nemegt Formation, Ankylosaurinae indet.					
ZPAL MgD I/43		620	557	187	1.11	0.34

^a^Specimens only preserve half of a tail club knob.

Medial to the third pair of caudal osteoderms is a transverse row of large scales (figure 6). These have the same black film as the osteoderm sheaths, and do not appear to contain bone, so we interpret these as epidermal scales rather than osteoderms with sheaths. Five large scales are arranged in one row, and two additional scales are located in a second posterior and slightly ventral row. They are flattened cones with basal widths of 50–65 mm, and posteriorly oriented, rounded apices. Isolated and less well-preserved epidermal scales are present at the base of the fourth pair of osteoderms, and at the anterior basal edge of the left osteoderm in the fifth pair.

## 8. Results of the phylogenetic analysis

**Figure 8.**
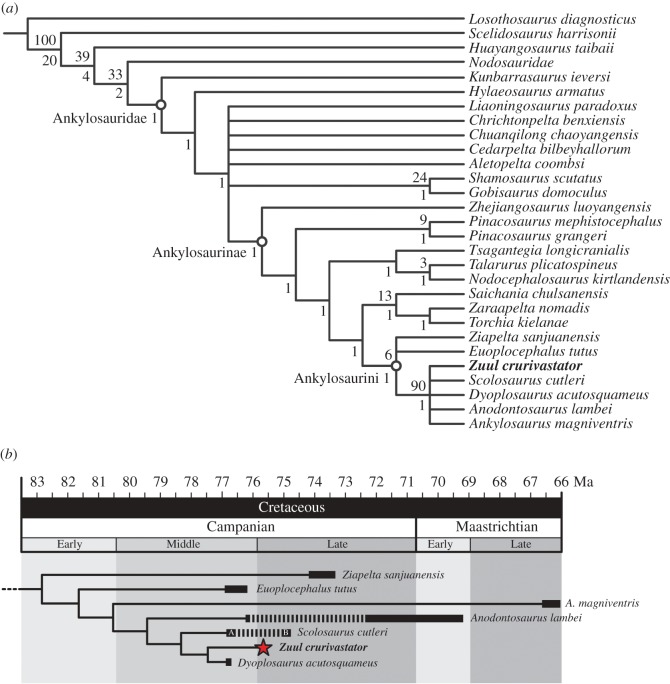
Results of the phylogenetic analysis. (*a*) Strict consensus of 10 most parsimonious trees (tree length = 563, consistency index = 0.403), with bootstrap values above nodes and Bremer support values below nodes. (A) 50% majority-rule tree of Ankylosaurini, showing approximate temporal ranges of species. Specimens of *Scolosaurus* are found in the Two Medicine Formation (*a*) and the lowest Dinosaur Park Formation (B). Specimens of *Anodontosaurus* are known from the Dinosaur Park Formation and the Horseshoe Canyon Formation.

The phylogenetic analysis recovered 10 most parsimonious trees, each with a tree length of 563, a consistency index of 0.403, and a retention index of 0.666 and a best tree-bisection reconnection score of 562 (figure 8). The strict consensus tree is reasonably well resolved overall with clear differentiation between Nodosauridae and Ankylosauridae, good resolution within Ankylosauridae and Ankylosaurinae, but relatively poor resolution within Ankylosaurini. *Nodocephalosaurus* was recovered outside of Ankylosaurini, in a clade with *Talarurus* and *Tsagantegia*, which, together, form the sister taxon to a clade containing *Saichania*, *Tarchia*, *Zaraapelta* and Ankylosaurini. *Zuul crurivastator* is recovered within Ankylosaurini, but the interrelationships within this clade are poorly resolved. *Euoplocephalus* and *Ziapelta* were resolved in a polytomy with a clade that includes *Ankylosaurus*, *Anodontosaurus*, *Dyoplosaurus*, *Scolosaurus* and *Zuul*, which were unresolved with respect to each other. Despite the seemingly poor resolution of these interrelationships, inspection of the 10 most parsimonious trees shows that the relative positions of *Ziapelta* and *Anodontosaurus* influence the overall topology within Ankylosaurini. In 9 of the 10 trees, *Zuul* is the sister taxon to *Dyoplosaurus* in a clade with *Scolosaurus*. In one tree, *Zuul* is instead the sister taxon to *Anodontosaurus*, which together are the sister clade to *Dyoplosaurus*. The 50% majority-rule tree (figure 8*b*) recovered *Zuul* as the sister taxon to *Dyoplosaurus*. Together, these were the sister taxon to a polytomy of *Scolosaurus* and *Anodontosaurus*, with *Ankylosaurus* as the next successive outgroup. *Ziapelta* and *Euoplocephalus* formed a polytomy that is the sister group to all other ankylosaurins.

## 9. Discussion

### 9.1. Comparison with other ankylosaurids

Morphological differences between ROM 75860 and other ankylosaurines allow it to be recognized as a distinct taxon, *Z. crurivastator.* The large size of the skull and body of ROM 75860 compared with other North American ankylosaurins (excluding *Ankylosaurus*; table 1) strongly suggests that this individual is skeletally mature. ROM 75860 is comparable in size (measured across the supraorbitals; table 1) to the largest individuals of *Euoplocephalus* (1.3% larger than ROM 1930 and 5.6% larger than AMNH 5405), *Anodontosaurus* (10.7% larger than TMP 1997.132.1 and 23% larger than CMN 8530), *Scolosaurus* (11% larger than MOR 433), *Dyoplosaurus* (10.7% larger than ROM 784) and *Ziapelta* (22% larger than NMMNH P-64484), and therefore, the proposed diagnostic features of *Zuul* are unlikely to represent ontogenetic variation within any existing ankylosaurin species. Relatively little is known about the growth trajectories of ankylosaurines. Osteohistological studies of ankylosaur limb elements by Stein *et al*. [40] found that even comparatively small individuals of *Scolosaurus* (NSM PV 20381) and *Anodontosaurus* (TMP 1982.9.3) had extensive secondary remodelling in the limbs, and the resulting absence of lines of arrested growth prohibited calculations of age at death. No limb elements have yet been identified and prepared for ROM 75860, and as such osteohistological assessments of the ontogenetic stage of this specimen are not yet possible.

*Zuul* is most similar to the Laramidian ankylosaurins *Anodontosaurus*, *Dyoplosaurus*, *Euoplocephalus*, *Scolosaurus* and *Ziapelta* [16,41,42]. These taxa all possess hexagonal, primarily flat frontonasal caputegulae and anterolaterally oriented external nares (figure 5). By contrast, Asian ankylosaurines such as *Saichania*, *Tarchia* and *Zaraapelta* [43–45] have strongly pyramidal frontonasal caputegulae, unlike the relatively flat caputegulae in *Zuul*. The rostrum of *Tarchia* and *Pinacosaurus grangeri* [20,34,43,46] is constricted anterior to the orbit, unlike the parallel-sided rostrum in *Zuul*. ROM 75860 also differs from the Laramidian *Nodocephalosaurus* and the Asian *Talarurus* [20,47–49], because these taxa have conical frontonasal caputegulae with circular bases. Additionally, *Nodocephalosaurus* has an anteriorly projecting, ridge-like loreal caputegulum unlike the low-relief loreal caputegulae in *Zuul* [20,49]. ROM 75860 is easily distinguished from *Ankylosaurus* because its external nares are anteriorly located rather than posteriorly located and ventrally directed [39]. *Zuul* also cannot be directly compared to the New Mexican taxon *Ahshislepelta* because of non-overlapping material at present; this ankylosaur was suggested to be an ankylosaurid by Burns and Sullivan [50] but recovered as a nodosaurid by Arbour and Currie [20]. *Zuul* is not referable to any known non-North American ankylosaurine, and therefore the remainder of our comparisons focus primarily on Laramidian ankylosaurins.

Numerous features of the skull indicate that ROM 75860 cannot be referred to *Ziapelta* (figure 5; [42]). *Ziapelta* has a large triangular median caputegulum, whereas *Zuul* has a small rectangular median nasal caputegulum. The frontonasal caputegulae in *Ziapelta* are gently bulbous, and in *Zuul* they are distinctly imbricated. *Ziapelta* has highly unusual squamosal horns that are laterally and ventrally curved, unlike the posteriorly directed squamosal horns of *Zuul* and other ankylosaurins. *Ziapelta* also has an unusual braincase morphology in which there are three longitudinal fossae on the basioccipital, which are absent in *Zuul*.

*Zuul* shares numerous traits with *Anodontosaurus*, *Euoplocephalus*, *Scolosaurus* and *Dyoplosaurus* (figure 5); for example, the anatomy of the palate and braincase of *Zuul* is indistinguishable from these taxa [16,42]. However, a suite of differences between ROM 75860 and *Anodontosaurus*, *Euoplocephalus*, *Scolosaurus* and *Dyoplosaurus* make it difficult to refer ROM 75860 to an existing species. Uniquely among ankylosaurines, the frontonasal caputegulae of *Zuul* are imbricated with distinct, posteriorly located peaks. The posterior frontonasal, prefrontal and middle supraorbital caputegulae of *Zuul* are also distinctly pyramidal, reminiscent of the caputegulae in Asian ankylosaurines like *Saichania*, *Tarchia* and *Zaraapelta* [43–45]. However, in ROM 75860, the pyramidal caputegulae are limited to the prefrontal and supraorbital regions, and they are smaller than in Asian ankylosaurines. None of the multiple specimens referred to *Dyoplosaurus*, *Euoplocephalus*, *Anodontosaurus* or *Scolosaurus* have tall, peaked caputegulae on the prefrontals or middle supraorbitals. Skulls referred to these species are either smooth or slightly rugose in this region [16,42]. *Zuul* also has distinct shallow apicobasal furrows on the lateral surfaces of the squamosal horns, which are not observed in *Ziapelta* or any of the multiple specimens of *Euoplocephalus*, *Anodontosaurus* or *Scolosaurus.*

Like *Anodontosaurus*, *Zuul* has postocular caputegulae. However, in *Zuul*, these are small, irregularly shaped, and sparsely distributed in the region behind the orbit, unlike the well-defined circular postocular caputegulae distributed along the base of the squamosal and quadratojugal horns in *Anodontosaurus* [16,51]. *Zuul* has a proportionately smaller median nasal caputegulum compared to *Anodontosaurus* (figure 5). *Anodontosaurus* has relatively short, pyramidal squamosal horns, which in dorsal view do not extend much past the nuchal shelf, and in lateral view are equilateral triangles with dorsally directed peaks. By contrast, the long squamosal horns of *Zuul* project far past the posterior margin of the skull in dorsal view, and in lateral view are shaped like isosceles triangles with posteriorly (rather than dorsally) directed apices.

Although there is only a partial skull known for *Dyoplosaurus* [16,52,53], the morphology of the ornamentation indicates that ROM 75860 is not referable to *Dyoplosaurus* (figure 5). The skull of ROM 784 preserves the prefrontal and supraorbital region, which lacks the prominent pyramidal caputegulae present in ROM 75860.

*Zuul* can be differentiated from *Euoplocephalus* based on the morphology of the squamosal horns, the size of the median nasal caputegulum, and the presence of postocular caputegulae (figure 5; [16,41]). *Euoplocephalus* has short pyramidal squamosal horns similar to *Anodontosaurus*, and ROM 75860 has long squamosal horns that project well past the nuchal shelf. The squamosal horns in *Euoplocephalus* also lack longitudinally oriented furrows. *Euoplocephalus* has a proportionately larger median nasal caputegulum, and lacks the postocular caputegulae that are present in *Zuul*.

*Scolosaurus* (including *Oohkotokia*, *sensu* Arbour and Currie [16]), like *Zuul*, has sparsely distributed, irregular postocular caputegulae (figure 5). It also has long, posteriorly projecting squamosal horns that in lateral view are isosceles triangles with posteriorly directed apices. However, *Scolosaurus* lacks the pyramidal prefrontal and middle supraorbital caputegulae, imbricated frontonasal caputegulae, and furrows on the squamosal horn, which are present in *Zuul*. The squamosal horn morphology is also subtly different between ROM 75860 and all known *Scolosaurus* skulls, as the lateral surface of the horn is convex in dorsal view and with a posteriorly pointing apex in all skulls of *Scolosaurus*, whereas the lateral edge is straight in dorsal view with a more laterally directed apex in ROM 75860. This difference, although subtle, appears to fall outside the range of variation represented by skulls of different sizes in *Scolosaurus*, and these features do not appear to vary substantially in *Euoplocephalus* ([16], figures 3–5); as such, the long, straight squamosal horn morphology in *Zuul* may be taxonomically significant. Although the holotype of *Scolosaurus* (NHMUK R5161) lacks a skull, *Zuul* can be differentiated from this specimen based on features of the postcranial osteoderms. NHMUK R5161 includes osteoderms in the medial and lateral positions on the tail (*sensu* Burns and Currie [38]), whereas medial caudal osteoderms appear to be absent in *Zuul*. The lateral caudal osteoderms in the anterior portion of the tail are more conical in NHMUK R5161 than ROM 75860, and the scalation pattern in the caudal region differs between these two specimens as well.

ROM 75860 is the only North American ankylosaurine skeleton to preserve substantial portions of the caudal integument *in situ*. ROM 1930 (*Euoplocephalus;* [16,54]), ROM 784 (*Dyoplosaurus;* [52,53]) and NHMUK R5161 (*Scolosaurus;* [54,55]) also preserve portions of the caudal integument, but the tail club is unknown for ROM 1930 and NHMUK R5161, and the integument in ROM 784 is not preserved on the tail club, except for the knob osteoderms. Isolated or associated tail clubs found in Alberta, Montana and Utah [16,56,57] lack osteoderms along the tail club handle (except for those forming the knob). By contrast, multiple specimens from Mongolia (PIN 614, MPC 100/1305, ZPAL MgD 1/III) preserve *in situ* caudal osteoderms (figure 7). The presence of osteoderms on the tail club handle in *Zuul*, and their absence in other ankylosaurins, can be interpreted in two ways: (i) the absence of caudal osteoderms on the tail club handle in most ankylosaurins is a taphonomic artefact, given that osteoderms are rarely found *in situ* over large parts of the body or (ii) ankylosaurins apomorphically lost the tail club handle osteoderms, which were convergently evolved in *Zuul*. A few lines of evidence suggest that their absence might be best attributed to taphonomy. ROM 784 preserves large triangular osteoderms just anterior to the tail club but not on the handle itself (figure 7, [53]). In this specimen, the ossified tendons, which would have lain deep to the osteoderms and are not found within the dermis or epidermis, are splayed laterally in the anterior part of the club. In many isolated tail clubs, and in ROM 75860, the ossified tendons are closely appressed to the handle vertebrae, and do not spread laterally. Additionally, osteoderms preserved in this specimen appear at least somewhat displaced: a conical osteoderm at the anterior edge of the tail club is preserved upside-down, and osteoderms are only preserved at the posterior end of the free caudal vertebrae. Together, this suggests that the integument on the tail had partly decomposed prior to fossilization, and thus their absence is taphonomic in origin.

The presence of lateral caudal osteoderms in Mongolian ankylosaurines, and general absence in North American ankylosaurines, may also reflect the highly disparate taphonomic settings of these two regions during the Late Cretaceous. Articulated dinosaur skeletons in the Djadokhta and Baruungoyot formations of Mongolia and China are often preserved in unusual positions, such as crouching [58,59], brooding nests of eggs [60,61], or locked in combat [58], which has been interpreted as evidence of entombment while the animal was still alive [58,62]. By contrast, many articulated dinosaur skeletons in Alberta are preserved in an opisthotonic ‘death pose’ with the head and neck retracted over the back (e.g. AMNH 5339, TMP 1991.36.500, TMP 1995.110.1), which represents entombment after death [63]. Mongolian ankylosaurines most likely had a higher chance of preserving the osteoderms *in situ* than Laramidian ankylosaurines, because they were entombed in place without fluvial action before or after death. ROM 75860 demonstrates that caudal osteoderms extended down the tail club handle in at least one genus of North American ankylosaurine. More specimens from North America are needed to assess whether or not these osteoderms were truly absent in other Laramidian ankylosaurins, but evidence from the differing taphomonic modes of Mongolian versus Laramidian ankylosaurines suggests that caution should be taken when inferring their presence or absence in Laramidian specimens.

The caudal osteoderms of *Zuul* can be compared to the Mongolian ankylosaurines MPC 100/1305 and PIN 614 (both cf. *Pinacosaurus;* [20,64–66]), and ZPAL MgD I/113 (Ankylosaurinae indet.; [67]) all of which preserve osteoderms along the length of the tail, including the tail club handle (figure 7). PIN 614 is mounted in ventral view with some of the caudal osteoderms left *in situ*; small ovoid osteoderms are present along the midline of the caudal centra, and a few paired, bluntly pointed osteoderms in the anterior part of the handle are ventrolaterally directed. Additional small, bluntly pointed osteoderms are mounted lateral to the tail club handle but it is unclear if these are *in situ*. MPC 100/1305 includes the best preserved caudal osteoderm series, and shows that pairs of osteoderms were arranged in dorsal, lateral and ventrolateral pairs along the tail, including the tail club. In MPC 100/1305, the dorsal osteoderms are typically keeled ovals, and the lateral and ventrolateral pairs are compressed, sharply pointed triangles, in dorsal view. The longest, most sharply pointed pair of lateral osteoderms are located at the anterior edge of the tail club handle, and posterior pairs become shorter; the penultimate pair anterior to the tail club knob are rounded rather than pointed. ZPAL MgD I/113 preserves sharply pointed triangular osteoderms lateral to the final three free caudal vertebrae, and lateral to the second and third handle vertebrae. Posteriorly on the handle, the osteoderms become mediolaterally shorter and have rounded lateral edges.

*Zuul* can be differentiated from Mongolian ankylosaurines based on the shape and arrangement of the caudal osteoderms (figure 7). ROM 75860 has five pairs of lateral osteoderms on the tail club (excluding the knob), compared to three in MPC 100/1305 [65,66]. Both taxa have sharply pointed, apicobasally long osteoderms in the anterior portion of the tail, which decrease in size and aspect ratio posteriorly along the tail club handle. *Zuul* also lacks the medial and ventrolateral osteoderms present in MPC 100/1305. Caudal osteoderms with a long, concave leading edge are unique to *Zuul*, as the caudal osteoderms in MPC 100/1305, PIN 614 and ZPAL MgD 1/111 [64–67] all have straight or slightly convex edges.

Arbour and Currie [16] argued that the proportions of the tail club knob were taxonomically informative for ankylosaurins. *Dyoplosaurus* has a narrow, elongate tail club knob, *Anodontosaurus* has a wide knob with pointed lateral edges, and *Euoplocephalus* and *Scolosaurus* have approximately circular knobs in dorsal view. *Zuul* is notable for having a particularly flattened tail club knob, which does not appear to be taphonomic in origin given the absence of obvious dorsoventral compaction in the tail club handle. ROM 75860 has a knob height to length ratio of 0.15, whereas most other tail club knobs have a ratio between 0.3 and 0.5. The asymmetry of the knob in *Zuul* (figures 6 and 7) is unusual but not without precedent in other specimens, as ROM 788 and UALVP 16247 (both isolated tail clubs from Dinosaur Provincial Park) are also notably asymmetric in dorsal view. Finally, a distinctive notch between the posterior corner of the major osteoderms and the minor osteoderms is present in *Zuul*, but also present in AMNH 5245 and AMNH 5216 (*Anodontosaurus*), and MACN Pv 12254 (an isolated club from the Dinosaur Park Formation). *Zuul* can be further distinguished from its close relative *Dyoplosaurus* based on the differing number of minor osteoderms and the absence of a sharp keel on the major osteoderms of the tail club knob.

ROM 75860 is the first ankylosaurid that preserves gular ossicles (figure 4). Gular ossicles have been reported in multiple specimens of *Stegosaurus stenops* [68,69] and the holotype of *Panoplosaurus mirus* (CMN 2759). In DMNH 2818 (*Stegosaurus*), the ossicles are square to hexagonal and range in diameter from 4 to 26 mm [69], and as such are generally substantially larger than those in *Zuul*. The ossicles in CMN 2759 (*Panoplosaurus*) include several very large, rounded ossicles as well as smaller ossicles comparable in size and morphology to those in *Zuul*. Neither *Stegosaurus* nor *Panoplosaurus* preserves the linear row of square gular ossicles found alongside the mandibular caputegulum in *Zuul*. In general, the gular ossicles in *Zuul* are consistent in morphology with the ossicles found in the thoracic, pelvic and caudal regions in other ankylosaurines [54]. Ossicles are also present along the tail all the way to the tail club knob, suggesting that a pavement of ossicles covered the entirety of the body in *Zuul*.

Skin impressions and preserved soft tissues, as in many other dinosaurs, are rarely encountered in ankylosaurids. Nevertheless, a few ankylosaurid specimens preserve integument that can be directly compared with that in ROM 75860 [54]. ROM 813 preserves a large swathe of integument from the pelvic region and possibly the anterior tail; one osteoderm preserves an impression of the overlying horny sheath [54]. Similar to ROM 75860, the surface texture of the horny sheath in ROM 813 does not match the surface texture of the underlying osteoderm. Although the osteoderm is smaller and of a different shape, both preserved sheaths show apicobasal striations and furrows at their bases.

The large epidermal scales on the dorsal surface of the tail are unique to ROM 75860 (figures 6 and 7). Epidermal scales in the caudal region of NHMUK R5161 (*Scolosaurus* [54,55]) are largest at the base of the osteoderms and markedly decrease in size away from the osteoderms. By contrast, the epidermal scales in ROM 75860 are arranged in a transverse row between the two large osteoderms. Additional preparation of the matrix covering the anterior portion of the tail may reveal more epidermal scales, and will help clarify the integument pattern in *Zuul* for comparisons with other ankylosaurins.

### 9.2. Implications for ankylosaurine evolution in western North America

The discovery of *Z. crurivastator* increases the known biodiversity of Laramidian ankylosaurins. Until recently, Laramidian ankylosaurin specimens were primarily assigned to three taxa: *E. tutus* and *Ankylosaurus magniventris* from northern Laramidia, and *Nodocephalosaurus kirtlandensis* from southern Laramidia [39,49,70,71]. Reviews of specimens referred to *Euoplocephalus* by Penkalski [56,72], Arbour *et al*. [53] and Arbour & Currie [16] demonstrated that these specimens represented at least three additional taxa, the previously named *A. lambei*, *D. acutosquameus* and *S. cutleri*. Arbour *et al*. [42] described a new southern Laramidian ankylosaur from New Mexico, *Ziapelta sanjuanensis*, and multiple as yet undescribed taxa have been collected from Utah [57]. Although less common in their communities (e.g. [73–75]), a higher diversity of ankylosaurins in the Campanian–Maastrichtian of Laramidia is consistent with the high morphological and taxic diversity observed in coeval megaherbivores, notably hadrosaurids and ceratopsids, yet these groups are still considerably more diverse [76–78].

The stratigraphic position of *Zuul* within the Coal Ridge Member of the Judith River Formation places it in a fauna that is much more poorly understood than that of the underlying McClelland Ferry Member [7]. The majority of species-level ornithischian records from the Judith River Formation originate from the McClelland Ferry Member [7], which was deposited during the regressive phase of the Western Interior Seaway [1]. Ornithischian taxa currently recognized from the McClelland Ferry Member include the pachycephalosaurid *Colepiocephale lambei* [79], the hadrosaurids *Brachylophosaurus canadensis* [80] and *Probrachylophosaurus bergei* [15], and the ceratopsids *Avaceratops lammersi* [81], *Judiceratops tigris* [82] and *Medusaceratops lokii* [83]. In addition, rich multi-taxic bonebeds have been excavated in this unit [4]. The dinosaurian fauna of the upper, transgressive unit of the formation, the Coal Ridge Member, is more poorly sampled for large dinosaur remains, but microvertebrate sites are common [6]. A reasonably complete centrosaurine ceratopsid skull previously referred to *A. lammersi* [81] is now considered an undiagnostic nasutoceratopsin [84]. Recently, two new chasmosaurine ceratopsids have been identified from the Coal Ridge Member, *Mercuriceratops gemini* [85] and *Spiclypeus shipporum* [7]. Lambeosaurine dinosaur nests have been described from the unit [86,87], but diagnostic hadrosaurid cranial material has not yet been described.

The Coal Ridge Member was deposited during the transgressive phase of the Judith River wedge, and as such it is broadly contemporaneous with the Dinosaur Park Formation of the Belly River Group of southern Alberta, which was deposited throughout the course of the same transgressive event [1,8,88]. The stratigraphically highest identifiable ankylosaurine in the Dinosaur Park Formation (ROM 1930) occurs 39 m above the base of the 80 m thick unit in Dinosaur Provincial Park [16,89], which corresponds to the onset of marine transgression in the Judith River wedge [8]. The host stratum of *Zuul* is estimated to occur at least 35 m above the base of the Coal Ridge Member and the corresponding onset of marine transgression, based on the 90 m thickness of the member in the Havre area ([1], fig. 12) and position of the *Zuul* quarry approximately 10 m below the Havre section presented by Eberth & Hamblin [27]. The base of the Coal Ridge Member has been radiometrically dated at 76.2 Ma, whereas a tuff in the middle of the Dinosaur Park Formation is dated at 76.39 Ma [90]. These dates are currently undergoing a major revision and are subject to change [91], but our best current estimate is that the *Zuul* quarry is slightly younger than the large sample of well-documented ankylosaurin specimens from the Dinosaur Park Formation, which are concentrated in the lower half of the unit [16,89]. Diagnostic ankylosaurin elements have not yet been recovered from lithostratigraphically and chronostratigraphically correlative strata of the upper muddy unit (unit 3) of the Oldman Formation across the US–Canada border to the north.

*Zuul* occurs stratigraphically lower, and definitively older, than the ankylosaurine specimens recovered from the Two Medicine Formation to the west [56]. Therefore, *Zuul* probably occurs within a chronostratigraphic gap in the northern Laramidian ankylosaurine fossil record between the known temporal range of *E. tutus* (*sensu* [16]) and *D. acutosquameus*, and the Two Medicine specimens. The skull of *Zuul* most closely resembles specimens assigned to *Scolosaurus* from the upper Two Medicine Formation because both taxa have long, backswept squamosal horns [16,56], and *Zuul* overlaps with the current chronostratigraphic range of *S. cutleri*. Results from the phylogenetic analysis showed a close relationship between *Zuul*, *Scolosaurus* and *Dyoplosaurus*. *Scolosaurus* is currently recognized from both the upper Two Medicine Formation and either the lowest Dinosaur Park Formation or uppermost Oldman Formation, representing a span of about 2 Myr [16], and *Dyoplosaurus* is recognized from the lower 30 m of the Dinosaur Park Formation [16]. The uppermost portion of the Two Medicine Formation represents a semi-arid, seasonal, well-drained alluvial floodplain, as does the Oldman Formation, in contrast with the more humid coastal plain environments represented by the Judith River [11,25] and Dinosaur Park formations [88]. The presence of distinct ankylosaurine species in close chronological and geographical proximity in northern Laramidia is intriguing given the different palaeoenvironments represented by the Two Medicine and Judith River formations, and suggests that closely related ankylosaurin species may have preferred different habitats.

In the past two decades, a renewed interest in the evolution, diversity dynamics and biogeography of Laramidian dinosaurs from the Campanian–Maastrichtian of western North America has resulted in multiple hypotheses regarding the evolutionary mechanisms underlying their morphological diversity, putative faunal provinciality and faunal turnover [12–15,42,89–100]. Arguments for provinciality have focused on ceratopsids [76], hadrosaurids [12,101], tyrannosaurids [102,103] and ankylosaurines [42]. New, undescribed taxa notwithstanding [57,104], assessment of provinciality in ankylosaurins is still difficult. Taxa resembling *Zuul* and its closest relatives *Scolosaurus* and *Dyoplosaurus* have not been discovered in southern Laramidia to date, nor have *Nodocephalosaurus* or *Ziapelta-like* taxa been found in Alberta or Montana, supporting the possibility of provinciality with the caveat that more dense temporal sampling is needed in coeval beds of northern and southern Laramidia to reasonably assess these hypotheses.

Anagenesis, or phyletic evolution within a single unbranching lineage, has also been implicated as generating considerable morphological diversity in ceratopsids [14] and hadrosaurids [14,15,94] without extensive speciation, yet to our knowledge has not been proposed in ankylosaurids due to poor sample size and inadequate stratigraphic control on the known specimens. An alternative interpretation of the *Zuul*, *Scolosaurus* and *Dyoplosaurus* clade is that at least some of this diversity reflects anagenesis rather than cladogenesis. *Zuul* occurs later than the known specimens of *Dyoplosaurus* from the lower Dinosaur Park Formation, and may be part of an anagenetic *Dyoplosaurus* lineage. *Zuul* occurs temporally between the holotype of *S. cutleri* from the middle Belly River Group of Alberta and specimens referred to this taxon from the upper Two Medicine Formation of Montana [16,56]. *Zuul* can easily be distinguished from each of these temporal endpoints, but it is possible that the cranial characters that distinguish *Zuul* from the Two Medicine *Scolosaurus* (=*O. horneri* [56]) and the postcranial characters that distinguish it from the *Scolosaurus* holotype may represent a combination of plesiomorphic and derived characters with respect to these temporal end members in an anagenetic lineage. More complete skeletons that preserve skulls, integument and tail clubs throughout the section are needed to test this hypothesis. The discovery of the remarkably complete skeleton of *Zuul* is, therefore, a key piece of data for understanding evolutionary patterns in Late Cretaceous armoured dinosaurs from Laramidia. Regardless of evolutionary mode, the ongoing refinement of ankylosaur biodiversity, biostratigraphy and phylogeny appears to document relatively higher rates of morphological evolution in this group during the Late Campanian than in the Maastrichtian, and is consistent with the pattern observed in other dinosaur groups [97,99,105].

## 10. Conclusion

ROM 75860 represents a new ankylosaurine genus and species, *Z. crurivastator* from the Judith River Formation. The holotype specimen is one of the most complete dinosaur skeletons from this formation, and is the first ankylosaurid skeleton described from this unit. Remarkably, ROM 75860 is the first ankylosaurin skeleton known with a complete skull and tail club, and it is the most complete ankylosaurid ever found in North America. The presence of abundant soft tissue preservation across the skeleton, including *in situ* osteoderms, skin impressions and dark films that probably represent preserved keratin, make this exceptional skeleton an important reference for understanding the evolution of dermal and epidermal structures in this clade. The recognition of *Z. crurivastator* from the Judith River Formation fills a gap in the ankylosaurine stratigraphic and geographical record and further highlights that Laramidian ankylosaurines were undergoing rapid evolutionary rates and stratigraphic turnover as observed for Laramidian ceratopsids, hadrosaurids, pachycephalosaurids and tyrannosaurids. The excellent preservation of ROM 75860 and the abundant diversity represented elsewhere in the same quarry highlight the potential for significant new fossil discoveries in the upper Judith River Formation and emphasize the need for continued work in this historically significant geological unit.

## Supplementary Material

Supporting Information 1

## Supplementary Material

Supporting Information 2

## Data accessibility

Electronic supplementary material, S1 and S2 are included as supplementary files at *Royal Society Open Science*. Additional high-resolution photographs of the skull, jaws and tail, and a video of the digital model of the skull and jaws, are available as supplementary materials at Dryad: http://dx.doi.org/10.5061/dryad.ts47s [106].
