# Supplementary material for: A new ankylosaurine dinosaur from the Judith River Formation of Montana, USA, based on an exceptional skeleton with soft tissue preservation
Source: R Soc Open Sci. 2017 May 10;4(5):161086. doi: 10.1098/rsos.161086 (PMC5451805; doi:10.1098/rsos.161086)
Supplement: Supporting Information 1 [file rsos161086supp1.docx]

**SUPPLEMENTARY INFORMATION 1: TAXA AND CHARACTER STATEMENTS**

Part 1: Specimens and references for taxa discussed in this study.

Part 2: Character statements used in the phylogenetic analysis.

Part 3: Literature cited.

**Part 1: Specimens and references for taxa discussed in this study.**

**Institutional abbreviations:** **AMNH** – American Museum of Natural History, New York, New York, USA; **BXGM** – Benxi Geological Museum, Liaoning, China; **CMN** – Canadian Museum of Nature, Ottawa, Ontario, Canada; **CEUM** – College of Eastern Utah Prehistoric Museum, Price, Utah, USA; **CYGYB** – Chaoyang Bird Fossil National Geopark, Chaoyang, Liaoning, China; **DMNH** – Denver Museum of Nature and Science, Denver, Colorado, USA; **FMNH** – Field Museum of Natural History, Chicago, Illinois, USA; **FWSHM** – Fort Worth Museum of Science and History, Fort Worth, Texas, USA; **IVPP** – Institute for Vertebrate Paleontology and Paleoanthropology, Beijing, China; **MLP** – Museo de La Plata, La Plata, Argentina; **MOR** – Museum of the Rockies, Bozeman, Montana, USA; **MPC** – Paleontological Center, Mongolian Academy of Sciences, Ulaanbaatar, Mongolia (MPC KID refers to Korea-Mongolia Dinosaur Project field numbers)**; MWC** – Museum of Western Colorado Dinosaur Journey, Fruita, Colorado, USA; **NHMUK** – Natural History Museum, London, United Kingdom; **NMMNH** – New Mexico Museum of Natural History and Science, Albuquerque, New Mexico, USA; **PIN** – Palaeontological Institute, Russian Academy of Sciences, Moscow, Russia; **ROM** – Royal Ontario Museum, Toronto, Ontario, Canada; **SDNHM** – San Diego Natural History Museum, San Diego, California, USA; **SMP** – State Museum of Pennsylvania, Harrisburg, Pennsylvania, USA; **SMU** – Shuler Museum of Paleontology, Southern Methodist University, Dallas, Texas, USA; **TMP** – Royal Tyrrell Museum of Palaeontology, Drumheller, Alberta, Canada; **UALVP** – University of Alberta Laboratory for Vertebrate Paleontology, Edmonton, Alberta, Canada; **USNM** – Smithsonian Museum of Natural History, Washington, DC, USA; **ZPAL** – Zaklad Paleobiologii, Polish Academy of Sciences, Warsaw, Poland.

***Ahshislepelta minor*:** SMP VP-1930; Burns and Sullivan (2011).

***Aletopelta coombsi*:** SDNHM 33909; Coombs and Deméré (1996), Ford and Kirkland (2001), Arbour and Currie (2016).

***Animantarx* *ramaljonesi*:** CEUM 6218, CEUM 6268, CEUM 8070, CEUM 8277, CEUM 8279, CEUM 8281, CEUM 9173; Carpenter et al. (1999).

***Ankylosaurus* *magniventris*:** AMNH 5895, AMNH 5214 (and skull cast UALVP 54722), CMN 8880.

***Anodontosaurus lambei*:** AMNH 5216, AMNH 5223, AMNH 5245, CMN 8530, NHMUK R4947, ROM 832, TMP 1982.9.3, TMP 1994.168.1, TMP 1996.75.01, TMP 1997.59.1, TMP 1997.132.01, USNM 10753; Arbour and Currie (2013).

**Argentinian ankylosaur:** MPCA-Pv 77, MPCA-Pv 68/69/70, MPCA-Pv 71, MPCA-Pv 72/73, MPCA-SM 1, MPCA-Pv 78, MPCA-Pv 41-43, 74-76; Coria and Salgado (2001).

***Bissektipelta archibaldi:*** Averianov (2002), Parish and Barrett (2004).

***Cedarpelta bilbeyhallorum:*** CEUM 1026, CEUM 10258, CEUM 10261, CEUM 10372, CEUM 10405, CEUM 10412, CEUM 10417, CEUM 10425, CEUM 10525, CEUM 10560, CEUM 10574, CEUM 12360, CEUM reconstructed cast skull; Carpenter et al. (2001).

***Chuanqilong chaoyangensis:*** Han et al. (2014).

***Crichtonpelta benxiensis:*** UALVP 52015 (cast of BXGMV0012), mounted skeleton on display at Sihetun Fossil Centre; Lü et al. (2007a), Arbour and Currie (2016).

***"Denversaurus" schlessmani:*** TMP 1993.145.0001; Bakker (1988), Carpenter (1990).

***Dongyangopelta yangyanensis:*** Chen et al. (2013).

***Dyoplosaurus acutosquameus:*** ROM 784, UALVP 47273; Arbour and Currie (2013).

***Edmontonia longiceps:*** CMN 8531; Sternberg (1928), Carpenter (1990).

***Edmontonia rugosidens:*** Gilmore (1930), Carpenter (1990).

***Euoplocephalus tutus:*** CMN 210, AMNH 5337, AMNH 5403, AMNH 5404, AMNH 5405, AMNH 5406, CMN 842, CMN 8876, ROM 1930, TMP 1979.14.74, UALVP 31, UALVP 47977; Arbour and Currie (2013).

***Europelta carbonensis:*** Kirkland et al. (2013).

***Gastonia burgei:*** CEUM 10293, CEUM cast pelvic shield, UALVP 54755 (cast of holotype skull), bonebed material, mostly caudals, at DMNH; Kirkland (1998).

***Gargoyleosaurus parkpinorum:*** DMNH 27726; Carpenter et al. (1998), Killbourne and Carpenter (2005).

***Gobisaurus domoculus:*** TMP 1990.000.4 (cast of holotype skull); Vickaryous et al. (2001), Arbour and Currie (2016).

***Hoplitosaurus marshi:*** USNM 4752; Lucas (1901), Carpenter and Kirkland (1998).

***Hungarosaurus tormai:*** Ősi (2005), Ősi and Makádi (2009), Ősi and Prondvai (2013), Ősi, Pereda Suberbiola, and Földes (2014).

***Hylaeosaurus armatus:*** NHMUK R3773, NHMUK R3782, NHMUK R3789, NHMUK R28936; Mantell (1833), Pereda-Suberbiola (1993).

***Kunbarrasaurus ieversi:*** USNM 508490; Molnar (1996), Molnar (2001), Leahey et al. (2015).

***Liaoningosaurus paradoxus:*** IVPP V12560, CYGYB 208, CYGYB 237; Xu et al. (2001), Arbour and Currie (2016).

***Mymoorapelta maysi:*** MWC holotype ilium, MWC 5819, MWC 1806, MWC 5820, MWC 5643, MWC 876, MWC 4027, MWC 3763, MWC 2610 block A4, MWC 5641, MWC 1801, MWC 1907, MWC 3744, MWC 5320, MWC 5435, MWC 5438; Kirkland and Carpenter (1994), Kirkland et al. (1998).

***Niobrarasaurus coleii:*** Mehl (1936), Carpenter et al. (1995); Everhart and Hamm (2005), Carpenter and Everhart (2007).

***Nodocephalosaurus kirtlandensis:*** SMP VP-900, Sullivan (1999), Arbour and Currie (2016).

***Nodosaurus textilis:*** Marsh (1889); Lull (1921), Carpenter and Kirkland (1998).

***Panoplosaurus mirus:*** CMN 2759; Lambe (1919), Carpenter (1990).

***Pawpawsaurus campbelli:*** FWMSH 93B.00026 (previously SMU 73203); Lee (1996), Carpenter and Kirkland (1998).

**Paw Paw scuteling:** FWMSH 93B.00025 (in part), SMU 72444 (in part); Jacobs et al. (1994).

***Peloroplites cedrimontanus:*** Carpenter et al. (2008).

***Polacanthus foxii:*** NHMUK R185, NHMUK R1926, NHMUK R4592, NHMUK R9293, cast of putative 'tail club' at CEUM; Blows (1987); Blows and Honeysett (2014).

***Pinacosaurus grangeri:*** AMNH 6523, MPC 100/1305, PIN 614, PIN 3780/3, ZPAL MgD II/1, ZPAL MgD II/9, ZPAL MgD II/31, MPC 100/1307, MPC 100/1308, MPC 100/1309, MPC 100/1310, MPC 100/1311, MPC 100/1312, MPC 100/1313, MPC 100/1315, MPC 100/1316, MPC 100/1317, manual elements; MPC 100/1318, MPC 100/1319, MPC 100/1320, MPC 100/1321, MPC 100/1322, MPC 100/1323, MPC 100/1324, MPC 100/1325, MPC 100/1326, MPC 100/1327, MPC 100/1328, MPC 100/1329, MPC 100/1330, MPC 100/1331, MPC 100/1332, MPC 100/1333, MPC 100/1334, MPC 100/1335, MPC 100/1335, MPC 100/1337, MPC 100/1338, MPC 100/1339, MPC 100/1340, MPC 100/1341, MPC 100/1342, MPC 100/1343, MPC 100/1344, MPC 100/1345, MPC 100/1346, MPC 100/1347, IVPP V16853, IVPP V16283, IVPP V16854, IVPP V16346, IVPP V16855; Arbour and Currie (2016).

***Pinacosaurus mephistocephalus:*** Godefroit et al. (1999).

***Saichania chulsanensis:*** Cast of holotype skull mounted with MPC 100/1305,cast of *in situ* holotype skeleton at ZPAL Museum of Evolution, PIN 3142/250; Maryańska (1977), Arbour and Currie (2016).

***Sauropelta edwardsorum:*** AMNH 3036; Ostrom (1970), Carpenter and Kirkland (1998)

***Sauroplites scutiger:*** AMNH 2074; Bohlin (1953).

***Scolosaurus cutleri:*** MOR 433, NHMUK R5161, TMP 2001.42.19, USNM 7943; Arbour and Currie (2013).

***Shamosaurus scutatus:*** PIN 3779/2; Tumanova (1983), Arbour and Currie (2016).

***Silvisaurus condrayi:*** Eaton (1960), Carpenter and Kirkland (1998).

***Stegopelta landerensis:*** FMNH UR88; Williston (1905), Moodie (1910), Carpenter and Kirkland (1998).

***Struthiosaurus austriacus:*** Pereda-Suberbiola and Galton (1994); Pereda Suberbiola and Galton (2001).

***Struthiosaurus languedocensis:*** Garcia and Pereda Suberbiola (2003).

***Struthiosaurus transylvanicus:*** NHMUK R4966; Nopcsa (1915).

***Talarurus plicatospineus:*** PIN 557; Maleev (1952).

***Taohelong jinchengensis:*** Yang et al. (2013).

***Tarchia kielanae:*** UALVP 49402 (cast of INBR 21004); Maryańska (1977), Arbour et al. (2014a).

***Tatankacephalus cooneyorum:*** Parsons and Parsons (2009).

***Texasetes pleurohalio:*** Coombs (1995), Carpenter and Kirkland (1998).

***"Tianchiasaurus nedegoapeferima":*** Dong (1993). (Not included in phylogenetic analysis)

***Tsagantegia longicranialis:*** MPC 700/17; Tumanova (1993), Arbour and Currie (2016).

***Zaraapelta nomadis:*** MPC D100/1388, Arbour et al. (2014a).

***"Zhejiangosaurus lishuiensis":*** Lü et al. (2007b), Arbour and Currie (2016).

***Ziapelta sanjuanensis:*** NMMNH P-64484, Arbour et al. (2014c).

***Zuul cruravastator:*** ROM 75860

**Part 2: Character statements used in the phylogenetic analysis.**

The character statements used in this analysis are identical to those published with Arbour and Currie (2016) and Arbour et al. (2016).

**Cranium**

1. Antorbital fenestra: present (0), absent (1). (Sereno 1999: 8, Thompson *et al.* 2012: 1).
2. Lateral temporal fenestra, visible in lateral view: visible (0), not visible (1). (Carpenter *et al.* 1998: 6; Thompson *et al.* 2012: 2).
3. Supratemporal fenestra: open (0), closed (1). (Lee 1996: 2, Thompson *et al.* 2012: 3)
4. Skull dimensions, including ornamentation: longer than wide (0), as wide, or wider than long (1). (Carpenter *et al.* 1998: 1, Thompson *et al.* 2012: 4)
5. Width of the posterior margin of the skull (including squamosal horns where applicable) relative to the maximum width across the orbits: greater or equal (0), less (1). (Vickaryous *et al.* 2004: 6, Thompson *et al.* 2012: 5).
6. Antorbital region of the dorsal skull surface: flat (0), arched (1). (Sereno 1999: 99; Thompson *et al.* 2012: 14, Arbour *et al.* 2014a: 10)
7. Deep longitudinal furrow on premaxilla: absent (0), present (1).
8. Ornamentation on premaxillary beak: absent (0), present (1).
9. Premaxillary sinus: absent (0), present (1).
10. Dimensions of premaxillary palate: longer than wide (0), wider than long (1). (Vickaryous *et al.* 2001: 13; Thompson *et al.* 2012: 18, Arbour *et al.* 2014a: 13).
11. Shape of the premaxillary palate: sub-triangular (0), sub-quadrate (1), sub-oval (2). (Sereno 1999: 80; Thompson *et al.* 2012: 19, Arbour *et al.* 2014a: 14)
12. ‘V’ or ‘U’-shaped median indentation of the anterior margin of the premaxilla: absent (0), present (1). (Sereno 1999: 91; Thompson *et al.* 2012: 20, Arbour *et al.* 2014a: 15).
13. Caudoventral extension of premaxillary tomium in lateral view: ends anteriorly to the maxillary teeth (0), obscures anteriormost maxillary teeth (1). (Sereno 1999: 100; Thompson *et al.* 2012: 21, Arbour *et al.* 2014a: 16).
14. Bone bordering anterior margin of internal nares: premaxilla (0), maxilla (1). (Thompson *et al.* 2012: 22, Arbour *et al.* 2014a: 17).
15. External nares, defined as the outermost rim of the nasal vestibule, opening faces: laterally to anterolaterally (0) anteriorly (1) ventrolaterally (2). (Carpenter *et al.* 1998: 10; Thompson *et al.* 2012: 7, Arbour *et al.* 2014a: 7)
16. External nares, visible in dorsal view: visible (0), hidden (1). (Thompson *et al.* 2012: 8, Arbour *et al.* 2014a: 8)
17. Paranasal apertures/fossae: no fossae or apertures present besides primary opening for nasal airway (0), paranasal apertures/fossae present (1). (After Hill et al. 2003, Sereno 1986, Coombs and Maryanska 1990).
18. Shape of respiratory passage: straight or arched (0), with anterior (rostral) and posterior (caudal) loops (*sensu* Witmer and Ridgely 2008; Arbour *et al.* 2014a: 148).
19. Vascular impressions on dorsal surface of posterior nasal passage (airway): absent (0) present (1). [After observations of vascular impressions in *Euoplocephalus* by Miyashita et al. 2011.]
20. Frontonasal and/or frontoparietal cranial ornamentation: absent (0) rugose, not differentiated into discrete polygons (caputegulae) (1), differentiated into discrete polygons (caputegulae) (2). (after Carpenter *et al.* 1999; Thompson *et al.* 2012: 78, Arbour *et al.* 2014a: 64).
21. Number of caputegulae in frontonasal and prefrontal region: no caputegulae (0), 10 or fewer (1), 11 to 30 (2) more than 30 (3). (Arbour *et al.* 2014c: 154)
22. Majority of frontonasal and/or frontoparietal caputegulum relief: caputegulae absent (0), caputegulae concave or flat (1), caputegulae strongly bulbous (2). (after Sullivan 1999 ; Thompson *et al.* 2012: 82, Arbour *et al.* 2014a: 67).
23. Domed frontonasal caputegulae: domed caputegulae absent (0), rounded cones with circular bases (1) pyramidal with sharp edges (2). (Arbour *et al.* 2014a: 150)
24. Supranarial caputegulae, notch or embayment dorsal to nasal vestibule: no supranarial caputegulae (0), notch absent (1), notch present (2).
25. Number of internarial caputegulae: none (0), 1 (1), more than 1 (2). (Arbour *et al.* 2014a: 151)
26. Median nasal caputegulum (located posterior to the supranarial ornamentation, on the midline of the skull), which is at least twice as large as the other frontonasal caputegulae: absent (0), present (1). (Vickaryous *et al.* 2004: 9, Thompson *et al.* 2012: 81, Arbour *et al.* 2014a: 66)
27. Loreal caputegulum in lateral view: no caputegulum (0) 1 caputegulum (1), more than 1 caputegulum (2). (Arbour *et al.* 2014a: 153)
28. Shape of the maxillary tooth row: straight (0), medially convex (1). (Vickaryous *et al.* 2001: 18; Thompson *et al.* 2012: 24, Arbour *et al.* 2014a: 18).
29. Maxillary tooth row position: lateral margin of skull (0), inset (1). (Lee 1996: 4; Thompson *et al.* 2012: 25, Arbour *et al.* 2014a: 20)
30. Distance between posteriormost extent of maxillary tooth rows relative to the width of the premaxillary beak: wider (0), narrower (1). [The width of the premaxillary beak is measured where the lateral edges of the beak are most parallel, which is usually close to the posterior of the premaxilla.] (Sereno 1999: 102; Thompson *et al.* 2012: 26, Arbour *et al.* 2014a: 21).
31. Caudoventral secondary palate: absent (0), present (1) (Vickaryous *et al.* 2004:21, Thompson *et al.* 2012: 49, Arbour *et al.* 2014a: 41)
32. Posterior palatal foramen: absent (0), present (1). (Lee 1996: 17; Thompson *et al.* 2012: 50, Arbour *et al.* 2014a: 42)
33. Gap between palate and braincase: open (0), closed by a dorsal projection of the pterygoid (1). (Sereno 1999: 61; Thompson *et al.* 2012: 16, Arbour *et al.* 2014a: 12).
34. Lacrimal incisure (mediolateral constriction behind the nares/at the prefrontals, giving the skull an hourglass-shaped outline in dorsal view): absent (0) present (1). (Arbour *et al.* 2014a: 149)
35. Lacrimal caputegulum in lateral view: no caputegulum (0) 1 caputegulum (1), more than 1 caputegulum (2). (Arbour *et al.* 2014a: 154)
36. Prefrontal caputegulum: no caputegulum (0) flat (1), sharply pointed and pyramidal (2). (Arbour *et al.* 2014a: 155)
37. Scroll-like descending process of the frontal: absent (0) present (1). [After observations of this feature in *Euoplocephalus* by Miyashita et al. 2011.]
38. Form of supraorbitals (including ornamentation): absent (0), boss-like, rounded laterally (1), sharp lateral rim, forming a ridge (2). (Vickaryous *et al.* 2001: 5; Thompson *et al.* 2012: 30, Arbour *et al.* 2014a: 23).
39. Supraorbitals, when viewed dorsally: no supraorbitals (0), combine to form continuous edge (1), have distinct apices (2). (Arbour *et al.* 2014a: 157)
40. Orbits, angle of orbital axis: <40º (0), >40º (1). (Thompson *et al.* 2012: 13, Arbour *et al.* 2014a: 9).
41. Ciliary osteoderm (eyelid ossification): absent (0) present (1)
42. Development of the postocular shelf: not developed (0), completely separating orbit from temporal space (1). (Sereno 1999: 104; Thompson *et al.* 2012: 15, Arbour *et al.* 2014a: 11)
43. Proportions of jugal orbital ramus: depth greater than transverse breadth (0), transverse breadth greater than depth (1). (Sereno 1999: 1; Thompson *et al.* 2012: 32, Arbour *et al.* 2014a: 24).
44. Depth of jugal ramus relative to orbit height: jugal height is less than 15% orbit height (0), jugal height is more than 15% orbit height (1).
45. Accessory postorbital ossification: absent (0), present (1) (Arbour *et al.* 2014a: 158)
46. Squamosal/postorbital horn: no horn (0) base has broad triangular cross-section and overall shape is pyramidal (1), base is oval in cross-section and overall shape is narrow, tapered cylinder (2). (Arbour *et al.* 2014a: 160)
47. Shape of jugal/quadratojugal horn in dorsal view: quadratojugal horn absent (0), horn U-shaped, with round distal edge (1), horn triangular, with pointed distal edge (2) (Thompson *et al.* 2012: 85, Arbour *et al.* 2014a: 71).
48. Jugal/quadratojugal horn: no horn (0) lacks distinct neck at base (1), has distinct neck at base (2).
49. Jugal or quadratojugal horn size relative to orbit size: no horn (0), length of base of jugal/quadratojugal horn equal to or less than the length of the orbit (1), length of base of jugal/quadratojugal horn is 110% or greater length of orbit (2).
50. Small (<2 cm diameter), circular caputegulae posterolateral to orbit (postocular caputegulae), along ventral edge of squamosal horn and/or along dorsal edge of quadratojugal horns: absent (0), present (1). (Arbour *et al.* 2014a: 142)
51. Form of the parietal surface: parietals flat to slightly convex (0), parietals concave, forming a trough-like surface posterior to the supraorbitals and anterior to the posterior edge of the skull (1). (Inverted from character 31 from Thompson *et al.* 2012)
52. A single large medial polygon of ornamentation in the parietal region: absent (0), present (1) (Thompson *et al.* 2012: 80, Arbour *et al.* 2014a: 65)
53. Number of discrete nuchal caputegulae: none (0), 2 (1), greater than 2(2)
54. Posterior projection of the nuchal shelf: does not obscure occiput in dorsal view (0), obscures occiput in dorsal view (1). (Vickaryous *et al.* 2004: 12, Thompson *et al.* 2012: 89, Arbour *et al.* 2014a: 73).
55. Shape of quadrate in lateral aspect: curved (anteriorly convex, posteriorly concave) (0), straight (1). (Vickaryous *et al.* 2001: 38; Thompson *et al.* 2012: 33, Arbour *et al.* 2014a: 25).
56. Inclination of quadrate in lateral aspect: near vertical (0), almost 45º anterolaterally (1). (Lee 1996: 10; Thompson *et al.* 2012: 34, Arbour *et al.* 2014a: 26).
57. Form of the anterior surface of the quadrate: transversely concave (0), not concave (1). (Lee 1996: 12; Thompson *et al.* 2012: 35, Arbour *et al.* 2014a: 27).
58. Ventral projection of the mandibular process of the quadrate in lateral view: projects beyond the quadratojugal ornamentation (0), hidden by quadratojugal ornamentation (1). (Vickaryous *et al.* 2004 : 40; Thompson *et al.* 2012: 36, Arbour *et al.* 2014a: 28).
59. Form of quadrate mandibular extremity: symmetrical (0), medial condyle larger than lateral condyle (1). (Sereno 1999: 10; Thompson *et al.* 2012: 37, Arbour *et al.* 2014a: 29).
60. Inclination of the articular surface of the quadrate condyle in posterior view: horizontal (0), ventromedially inclined at approximately 45° to horizontal (1). (Sereno 1999: 14; Thompson *et al.* 2012: 38, Arbour *et al.* 2014a: 30).
61. Lateral ramus of the quadrate: present (0), absent (1). (Sereno 1999: 15; Thompson *et al.* 2012: 39, Arbour *et al.* 2014a: 31).
62. Dorsoventral depth of the pterygoid process of the quadrate: deep (0), shallow (1). (Lee 1996: 7; Sereno 1999: 60; Thompson *et al.* 2012: 40, Arbour *et al.* 2014a: 32).
63. Contact between paroccipital process and quadrate: sutural (0), fused (1). (Carpenter *et al.* 1998: 13; Thompson *et al.* 2012: 41, Arbour *et al.* 2014a: 33).
64. Contact between pterygoids: pterygoids separate caudomedially, forming an interpterygoid vacuity (0), pterygoids joined medially forming a pterygoid shield (1). (Thompson *et al.* 2012: 42, Arbour *et al.* 2014a: 34).
65. Direction of the pterygoid flange: anterolateral (0), anterior/parasagittal (1). (Vickaryous *et al.* 2001: 29; Thompson *et al.* 2012: 43, Arbour *et al.* 2014a: 35).
66. Contact between basipterygoid processes and pterygoid: sutural (0), fused (1). (Vickaryous *et al.* 2001: 30; Thompson *et al.* 2012: 44, Arbour *et al.* 2014a: 36).
67. Position of ventral margin of the pterygovomerine keel relative to alveolar ridge: dorsal (0), level (1). (Sereno 1999: 59; Thompson *et al.* 2012: 45, Arbour *et al.* 2014a: 37).
68. Dorsal extent of median vomer lamina: does not meet skull roof (0), meets skull roof (1). (Lee 1996: 14; Thompson *et al.* 2012: 46, Arbour *et al.* 2014a: 38).
69. Pterygoid foramen: absent (0), present (1). (Hill *et al.* 2003: 21; Thompson *et al.* 2012: 47, Arbour *et al.* 2014a: 39).
70. Position of posterior margin of pterygoid body relative to the anterior margin of the quadrate condyle: anteriorly positioned (0), in transverse alignment (1). (Vickaryous *et al.* 2004: 28, Thompson *et al.* 2012: 48, Arbour *et al.* 2014a: 40).
71. Size of occiput: higher than wide (0), wider than high (1). (Sereno 1999: 99, Thompson *et al.* 2012: 6, Arbour *et al.* 2014a: 6).
72. Direction of paroccipital process extension: caudolateral (0), lateral (1). (Carpenter *et al.* 1998: 11; Vickaryous *et al.* 2004: 33 ; Thompson *et al.* 2012: 51, Arbour *et al.* 2014a: 43).
73. Bones forming the occipital condyle: basioccipital and exoccipital (0), basioccipital only (1). (Lee 1996: 9; Thompson *et al.* 2012: 54, Arbour *et al.* 2014a: 44).
74. Length of basisphenoid relative to the basioccipital: longer (0), shorter or equal (1). (Sereno 1999: 12; Thompson *et al.* 2012: 56, Arbour *et al.* 2014a: 45).
75. Form of basisphenoidal tuberosities: medially separated rounded rugose stubs (0), continuous transverse rugose ridge (1). (Vickaryous *et al.* 2001: 32; Thompson *et al.* 2012: 57, Arbour *et al.* 2014a: 46).
76. Size of basipterygoid processes: twice as long as wide or over (0), less than twice as long as wide (1). (Thompson *et al.* 2012: 58, Arbour *et al.* 2014a: 47).
77. Form of the cranial nerve foramina IX-XII: separate foramina (0), single foramen shared with the jugular vein (1). (Thompson *et al.* 2012: 59, Arbour *et al.* 2014a: 48).

Mandibles

1. Position of mandible articulation relative to mandibular adductor fossa: posterior (0), posteromedial (1). (Sereno 1999: 64; Thompson *et al.* 2012: 67, Arbour *et al.* 2014a: 55).
2. Mandibular fenestra: present (0), absent (1). (Thompson *et al.* 2012: 68).
3. Depth of the dentary symphysial ramus relative to half the maximum depth of the mandibular ramus in lateral view: deeper (0), shallower (1). (Sereno 1999: 17; Thompson *et al.* 2012: 69, Arbour *et al.* 2014a: 57).
4. Shape of dorsal margin of the dentary in lateral view: straight (0), sinuous (1). (Sereno 1999: 4; Thompson *et al.* 2012:70, Arbour *et al.* 2014a: 58).
5. Development of the coronoid process: not developed (0), distinct (1). (Sereno 1999: 108; Thompson *et al.* 2012: 73, Arbour *et al.* 2014a: 59).
6. Position of glenoid for quadrate relative to mandibular axis: medially offset (0), in line (1). (after Carpenter *et al.* 1999; Thompson *et al.* 2012: 74, Arbour *et al.* 2014a: 60).
7. Size and projection of the retroarticular process: small with no dorsal projection (0), well developed with a dorsal projection (1). (Thompson *et al.* 2012: 75, Arbour *et al.* 2014a: 61).
8. Size of predentary ventral process: distinct, prong shaped process (0), rudimentary eminence (1). (Sereno 1999: 66; Thompson *et al.* 2012:76, Arbour *et al.* 2014a: 62).
9. Length of mandibular caputegulum with respect to the length of the mandible: no caputegulum (0), less than or equal to half the length (1), over three quarters the length (2). (after Carpenter *et al.* 1999; Thompson *et al.* 2012: 90, Arbour *et al.* 2014a: 74).

Teeth

1. Premaxillary teeth: present (0), absent (1). (Sereno 1999: 18; Thompson *et al.* 2012: 63, Arbour *et al.* 2014a: 51).
2. Cingula on maxillary and/or dentary teeth: absent (0), present (1). (Carpenter *et al.* 1998: 21; Thompson *et al.* 2012: 64, Arbour *et al.* 2014a: 52).
3. Maxillary and/or dentary tooth crown shape: pointed (0), rounded (1). (Thompson *et al.* 2012: 65, in part; Arbour *et al.* 2014a: 53)
4. Maxillary and/or dentary tooth denticles: < 13 denticles (0), ≥13 denticles (1) (Thompson *et al.* 2012: 65, in part; Arbour *et al.* 2014a: 53).
5. Number of dentary teeth: <25 (0), ≥25 (1). (Thompson *et al.* 2012: 66, Arbour *et al.* 2014a: 54).

Axial Skeleton

1. Type of articulation between the atlantal neural arch and intercentrum: open (0), fused in adult (1). (Sereno 1999: 19; Thompson *et al.* 2012: 92, Arbour *et al.* 2014a: 76).
2. Type of contact between the atlantal neural arches: no median contact (0), median contact (1). (Sereno 1999: 68; Thompson *et al.* 2012: 93, Arbour *et al.* 2014a: 77).
3. Contact between atlas and axis: articulated (0), fused (1). (Vickaryous *et al.* 2004: 46; Thompson *et al.* 2012: 94, Arbour *et al.* 2014a: 78).
4. Dimensions of cervical vertebrae centra: anteroposteriorly longer than transverse width (0), anteroposteriorly shorter than transverse width (1). (after Kirkland *et al.* 1998; Thompson *et al.* 2012: 95, Arbour *et al.* 2014a: 79).
5. Ratio of maximum neural spine width to height in anterior cervicals: <0.25 (0), ≥0.25 (1). (after Carpenter *et al.* 1999; Thompson *et al.* 2012: 96, Arbour *et al.* 2014a: 80).
6. Ratio of anteroposterior dorsal centrum length to posterior centrum height: >1.1 (0), <1.1 (1). (Thompson *et al.* 2012: 98, Arbour *et al.* 2014a: 82).
7. Longitudinal keel on ventral surface of dorsal centra: present (0), absent (1). (Thompson *et al.* 2012: 99, Arbour *et al.* 2014a: 83).
8. Cross sectional shape of neural canal in posterior dorsals: circular (0) elliptical, with long axis running dorsoventrally (1). (after Carpenter 1990; Thompson *et al.* 2012: 100, Arbour *et al.* 2014a: 84).
9. Shape of the proximal cross-section of the dorsal ribs: triangular (0), ‘L’- or ‘T’-shaped (1). (Thompson *et al.* 2012: 101, Arbour *et al.* 2014a: 85)
10. Attachment of dorsal ribs to posterior dorsal vertebrae: articulated (0), fused (1). (Thompson *et al.* 2012: 102, Arbour *et al.* 2014a: 86).
11. Contact between posteriormost dorsal vertebrae: articulated (0), fused to form a presacral rod (1). (Thompson *et al.* 2012: 103, Arbour *et al.* 2014a: 87).
12. Longitudinal groove in ventral surface of the sacrum: absent (0), present (1). (Thompson *et al.* 2012: 105, Arbour *et al.* 2014a: 89).
13. Longitudinal ridge at approximate mid-height of centrum of mid and distal caudals: absent (0) present (1).
14. Ratio of maximum distal width to height of the neural spines of proximal caudals: ≤0.2 (0), >0.2 (1). (after Carpenter 2001, Thompson *et al.* 2012: 107).
15. Direction of the transverse processes of proximal caudals: craniolaterally projecting (0), caudolaterally projecting (1), laterally projecting (2). (after Carpenter 2001; Thompson *et al.* 2012: 107, Arbour *et al.* 2014a: 90).
16. Persistence of transverse processes down the length of the caudal series: not present beyond the mid-length of the series (0), present beyond the mid-length of the series (1). (Thompson *et al.* 2012: 110, Arbour *et al.* 2014a: 92).
17. Attachment of haemal arches to their respective centra: articulated (0), fused (1). (Thompson *et al.* 2012: 111, Arbour *et al.* 2014a: 93).
18. Extent of pre- and postzygapophyses over their adjacent centra in posterior vertebrae: extend over less than half the length of the adjacent centrum (0), extend over more than half the length of the adjacent centrum (1). (Sereno 1999: 109; Thompson *et al.* 2012:113, Arbour *et al.* 2014a: 95).
19. In tail club handle vertebrae, shape of each interlocking neural arch in dorsal view: distal caudal vertebrae do not form handle (0), V-shaped, angle of divergence about 22-26° (1), V-shaped, angle of divergence about 35-37° (2), U-shaped, angle of divergence greater than 60° (3).
20. Shape of the posterior haemal arches: rounded haemal spine in lateral view with no contact between haemal arches (0), inverted ‘T’-shaped haemal spine in lateral view, with contact between the ends of adjacent spines (1). (Sereno 1999: 71; Thompson *et al.* 2012: 114, Arbour *et al.* 2014a: 96).
21. Ossified tendons in distal region of tail: absent (0), present (1). (Sereno 1999: 97; Thompson *et al.* 2012: 115, Arbour *et al.* 2014a: 97).

Pectoral Girdle

1. Dimensions of coracoid: longer than wide (0), wider than long or equal width and length (1). (Thompson *et al.* 2012: 116, Arbour *et al.* 2014a: 98).
2. Form of the anterior margin of the coracoid: convex (0), straight (1). (Thompson *et al.* 2012: 117, Arbour *et al.* 2014a: 99).
3. Cranioventral process of coracoid: absent (0), present (1). (Thompson *et al.* 2012: 118, Arbour *et al.* 2014a: 100).
4. Size of coracoid glenoid relative to scapula glenoid: sub-equal (0), half the size (1). (Sereno 1999: 89; Thompson *et al.* 2012: 119, Arbour *et al.* 2014a: 101).
5. Contact between scapula and coracoid: articulated (0), fused (1). (Thompson *et al.* 2012: 120, Arbour *et al.* 2014a: 102).
6. Scapula glenoid orientation: ventrolateral (0), ventral (1). (Sereno 1999: 87; Thompson *et al.* 2012: 121, Arbour *et al.* 2014a: 103).
7. Ventral process of scapula at the caudoventral margin of glenoid: absent (0), present (1). (Thompson *et al.* 2012: 122, Arbour *et al.* 2014a: 104).
8. Form of the scapula acromion process: not developed or ridge-like along the dorsal border of the scapula (0) flange-like and folded over towards the scapula glenoid (1) ridge terminating in a knob-like eminence (2). (Vickaryous *et al.* 2004: 52, Thompson *et al.* 2012: 123, Arbour *et al.* 2014a: 105)
9. Orientation of the acromion process of scapula: directed away from the glenoid (0), directed towards scapula glenoid (1). (after Kirkland 1998; Thompson *et al.* 2012: 124, Arbour *et al.* 2014a: 106)
10. Scapulocoracoid buttress: absent (0), present (1). (Thompson *et al.* 2012: 125, Arbour *et al.* 2014a: 107).
11. Distal end of scapula shaft: narrow (0), expanded (1). (Sereno 1999: 20; Thompson *et al.* 2012: 126, Arbour *et al.* 2014a: 108).
12. Contact between sternal plates: separate (0), fused (1). (Sereno 1999: 112; Vickaryous *et al.* 2004: 60; Thompson *et al.* 2012: 127, Arbour *et al.* 2014a: 109).

Pelvic Girdle

1. Length of the preacetabular process of ilium as a percentage of total ilium length: ≤ 50% (0), > 50 % (1).(Thompson *et al.* 2012: 136, Arbour *et al.* 2014a: 118).
2. Angle of lateral deflection of the preacetabular process of the ilium: 10º–20º (0), 45º (1). (Sereno 1999: 21; Thompson *et al.* 2012: 137, Arbour *et al.* 2014a: 119).
3. Orientation of the preacetabular portion of the ilium: near vertical (0), near horizontal (1). (Kirkland 1998: 45; Thompson *et al.* 2012: 138, Arbour *et al.* 2014a: 120).
4. Form of the preacetabular portion of the ilium: straight process (0), pronounced ventral curvature (1). (Thompson *et al.* 2012: 139, Arbour *et al.* 2014a: 121).
5. Lateral edge of ilium in dorsal view: straight (0), sinuous (1).
6. Lateral exposure of the acetabulum: exposed (0) acetabulum partially obscured as it is partially encircled by the distal margin of the ilium (1). (Thompson *et al.* 2012: 140, Arbour *et al.* 2014a: 122)
7. Perforation of the acetabulum: present, open acetabulum (0), absent, closed acetabulum (1). (Sereno 1999: 74; Thompson *et al.* 2012: 141, Arbour *et al.* 2014a: 123).
8. Postacetabular ilium length, relative to diameter of acetabulum: greater (0), smaller (1). (Sereno 1999: 114; Thompson *et al.* 2012: 142, Arbour *et al.* 2014a: 124).
9. Pubis: present (0), indistinct from ilium/ischium (1) (Kirkland 1998: 46, Thompson *et al.* 2012: 143)
10. Shape of ischium: straight (0), ventrally flexed at mid-length (1). (Kirkland 1998: 37; Thompson *et al.* 2012: 147, Arbour *et al.* 2014a: 125).
11. Shape of the dorsal margin of ischium: straight or concave (0), convex (1). (Sereno 1999: 115; Thompson *et al.* 2012: 148, Arbour *et al.* 2014a: 126).

Limbs

1. Separation of humeral head and deltopectoral crest in anterior view: continuous (0), separated by a distinct notch (1). (Thompson *et al.* 2012: 128, Arbour *et al.* 2014a: 110).
2. Separation of humeral head and medial tubercle in anterior view: continuous (0), separated by a distinct notch (1) (Thompson *et al.* 2012: 129, Arbour *et al.* 2014a: 111).
3. Ratio of deltopectoral crest length to humeral length: ≤0.5 (0), >0.5 (1). (Thompson *et al.* 2012: 130, Arbour *et al.* 2014a: 112).
4. Orientation of deltopectoral crest projection: lateral (0), anterolateral (1). (Sereno 1999: 113; Thompson *et al.* 2012: 131, Arbour *et al.* 2014a: 113).
5. Shape of the radial condyle of humerus round / proximal end of radius in end-on view: non-circular (0), circular (1). (Thompson *et al.* 2012: 132, Arbour *et al.* 2014a: 114).
6. Ratio of the length of metacarpal V to metacarpal III: ≤0.5 (0), >0.5 (1). (Sereno 1999: 6; Thompson *et al.* 2012: 133, Arbour *et al.* 2014a: 115).
7. Manual digit number: 5 (0), 4 (1), 3 (2). (Thompson *et al.* 2012: 134, Arbour *et al.* 2014a: 116).
8. Shape of manual and pedal ungual phalanges: narrow, claw-shaped (0) wide, hoof-shaped, U-shaped in dorsal view (1), wide, hoof-shaped, triangular in dorsal view (2) (Sereno 1999: 7; Thompson *et al.* 2012: 135, Arbour *et al.* 2014a: 117).
9. Angle between long axis of femoral head and long axis of shaft: <100º (0), 100º to 120º (1), >120º (2). (Thompson *et al.* 2012: 149, Arbour *et al.* 2014a: 127).
10. Separation of femoral head from greater trochanter: continuous (0), separated by a distinct notch or change in slope (1). (Thompson *et al.* 2012: 150, Arbour *et al.* 2014a: 128).
11. Differentiation of the anterior trochanter of the femur: separated from femoral shaft by a deep groove laterally and dorsally (0), fused to femoral shaft (1). (Kirkland 1998: 36; Thompson *et al.* 2012: 151, Arbour *et al.* 2014a: 129).
12. Oblique ridge on lateral femoral shaft, distal to anterior trochanter: absent (0), present (1). (Thompson *et al.* 2012: 152, Arbour *et al.* 2014a: 130).
13. Form of the fourth trochanter: pendant (0), ridge-like (1). (Sereno 1999: 24; Thompson *et al.* 2012: 153, Arbour *et al.* 2014a: 131).
14. Location of the fourth trochanter on the femoral shaft: proximal (0) distal, over half-way down the femoral shaft (1). (Thompson *et al.* 2012: 154, Arbour *et al.* 2014a: 132).
15. Maximum distal width of the tibia, compared to the maximum proximal width: narrower (0), wider (1). (Sereno 1999: 188; Thompson *et al.* 2012: 155, Arbour *et al.* 2014a: 133).
16. Contact between tibia and astragalus: articulated (0), fused, with suture obliterated (1). (Thompson *et al.* 2012: 156, Arbour *et al.* 2014a: 134).
17. Number of pedal digits: 5 (0), 4 (1), 3 (2). (Thompson *et al.* 2012: 157, Arbour *et al.* 2014a: 135).
18. Phalangeal number in pedal digit IV: 5 (0), ≤4 (1). (Sereno 1999: 26; Thompson *et al.* 2012: 158, Arbour *et al.* 2014a: 136).

Postcranial osteoderms and integument

1. Postcranial osteoderm distribution: no postcranial osteoderms (0); postcranial osteoderms arranged in multiple transverse rows (1); postcranial osteoderms primarily present in two rows along midline (2). (Modified from Thompson *et al.* (2012) characters 159-161, and Burns and Currie 2014: 69).
2. Dimensions of largest osteoderm: no osteoderms (0) smaller than a dorsal centrum (1), equal to or larger than a dorsal centrum (2). (Lee 1997: 125, Hill 2005: 309, Burns and Currie 2014: 68)
3. Basal surface of osteoderms: no osteoderms (0) flat or gently concave (1), deeply excavated (2), strongly convex (3). (Carpenter 2001:34, Hill 2005:331, Burns and Currie 2014:72).
4. External cortical histology of skeletally mature osteoderms: no osteoderms (0) lamellar bone (1), ISFB (2). (Burns and Currie 2014: 80)
5. Haversian bone in osteoderms: no osteoderms (0) absent in core of skeletally mature osteoderms (1), may be present in in core of skeletally mature osteoderms (2). (Burns and Currie 2014: 81)
6. Basal cortex of skeletally mature osteoderms: no osteoderms (0) present (1), absent or poorly developed (2). (Burns and Currie 2014: 82)
7. Structrural fiber arrangement in osteoderms: no osteoderms (0) structural fibres absent (1), reaches orthoganal arrangment near osteoderm surfaces (2), diffuse throughout (3), highly ordered sets of orthogonally arranged fibers in the superficial cortex (4). (Burns and Currie 2014: 91)
8. Gular osteoderms: absent (0), present (1). (Hill 2005:305; Burns and Currie 2014: 65)
9. Number of distinct cervical pectoral bands: none (0), one (1), two (2). (Kirkland 1998: 38; Thompson *et al.* 2012: 162, Arbour *et al.* 2014a: 138)
10. Form of cervical half rings: cervical half rings absent (0), composed of osteoderms that are either tightly adjacent to one another or coossified at the edges, forming arc over the cervical region (1), composed of osteoderms and underlying bony band segments, osteoderms may or may not cossify to the band, forming arc over the cervical region (2). (Arbour *et al.* 2014a: 143)
11. Composition of first cervical half ring with band: no cervical half ring with band (0), first cervical half ring has 4 to 6 primary osteoderms only (1), first cervical half ring has 4 to 6 primary osteoderms surrounded by small (<2 cm diameter) circular secondary osteoderms (2). (Arbour *et al.* 2014a: 144)
12. Distal spines on cervical half ring: absent (0), present, projecting dorsoposteriorly (1), present, projecting anteriorly (2). (Carpenter 2001: 83, Burns and Currie 2014: 85)
13. Osteoderms on proximal limb segments: absent (0), present (1). (deBraga and Rieppel 1997:167, Lee 1997: 127, Heckert and Lucas 1999:60, Hill 2005: 306, Burns and Currie 2014: 66)
14. Millimeter-sized ossicles abundant in spaces between osteoderms in thoracic or caudal regions (excluding pelvic region), absent (0), present (1). [Based on observations presented in Arbour et al. (2014b)]
15. Deeply excavated, dorsoventrally flattened triangular osteoderms: absent (0), right or obtuse-angled triangles (1), right or obtuse-angled triangles that abruptly narrow distally into a spike ('splates' of Blows 2001) (2).
16. On deeply excavated triangular osteoderms, furrows perpendicular to basal edge: no deeply excavated triangular osteoderms (0), furrows absent (1), furrows present (2)
17. Lateralmost osteoderms in thoracic region: absent (0), ovoid or sub-ovoid with a longitudinal keel (1) triangular, dorsoventrally flattened elements (2), solid, conical spikes (3). (Carpenter 2001:36, Hill 2005: 336, Burns and Currie 2014: 73)
18. Thoracic osteoderms coossified to dorsal ribs: no osteoderms coossified to ribs (0), at least some osteoderms coossified to ribs (1)
19. Form of pelvic osteoderms: no osteoderms (0) unfused (1), coossified osteoderm rosettes (2), coossified evenly-sized polygons (3). (Arbour *et al.* 2014a: 140)
20. Caudal osteoderms: absent (0), present on dorsal or dorsolateral surfaces of tail only (1), completely surrounding tail (2). (Hill 2005:307, Burns and Currie 2014: 67)
21. Morphology of proximal, lateral caudal osteoderms: osteoderms absent (0), triangular with round/blunt apex (1) triangular with pointed apex (2). (Hill 2005:342, Burns and Currie 2014: 75)
22. Keel height of caudal osteoderms relative to thoracic osteoderms: osteoderms absent (0), keels equal in external-basal height (1), keels taller in caudal osteoderms (2). (Hill 2005: 343, Burns and Currie 2014: 76)
23. Tail club knob shape: knob absent (0), major knob osteoderms semicircular in dorsal view (1), triangular in dorsal view (2). (Arbour & Currie 2013:175)
24. Tail club knob proportions: knob absent (0), tail club knob length > width (1), length = width (2), width > length (3) (Arbour & Currie 2013:176)

**Part 3: Literature cited:**

Arbour VM, Currie PJ. 2016. Systematics, phylogeny and palaeobiogeography of the ankylosaurid dinosaurs. Journal of Systematic Palaeontology 14: 385-444.

Arbour VM, Zanno LE, Gates TA. 2016. Ankylosaurian dinosaur palaeoenvironmental associations are influenced by sea level fluctuation, extirpation, and geodispersal. Palaeogeography, Palaeoclimatology, Palaeoecology 449:289-299.

Arbour VM, Currie PJ. 2013. *Euoplocephalus tutus* and the diversity of ankylosaurid dinosaurs in the Late Cretaceous of Alberta, Canada, and Montana, USA. PLOS ONE 8: e62421.

Arbour VM, Currie PJ, Badamgarav D. 2014a. The ankylosaurid dinosaurs of the Upper Cretaceous Baruungoyot and Nemegt formations of Mongolia. Zoological Journal of the Linnean Society 172: 631-652.

Arbour VM, Burns ME, Bell PR, Currie PJ. 2014b. Epidermal and dermal integumentary structures of ankylosaurian dinosaurs. Journal of Morphology 275: 39-50.

Arbour VM, Burns ME, Sullivan RM, Lucas SG, Cantrell AK, Fry J, Suazo TL. 2014c. A new ankylosaurid dinosaur from the Upper Cretaceous (Kirtlandian) of New Mexico, with implications for ankylosaurid diversity in the Upper Cretaceous of western North America. PLOS ONE 9: e108804.

Averianov AO. 2002. An ankylosaurid (Ornithischia: Ankylosauria) from the Upper Cretaceous Bissekty Formation of Uzbekistan. Bulletin de l'Institut Royal des Sciences Naturelles de Belgique, Sciences de la Terre 72: 97-110.

Bakker RT. 1988. Review of the Late Cretaceous nodosauroid Dinosauria – *Denversaurus schlessmani*, a new armor-plated dinosaur from the latest Cretaceous of South Dakota, the last survivor of the nodosaurians, with comments on stegosaur-nodosaur relationships. Hunteria 1:1-21.

Blows WT. 1987. The armoured dinosaur *Polacanthus foxi* from the Lower Cretaceous of the Isle of Wight. Palaeontology 30:557-580.

Blows WT. 1996. A new species of *Polacanthus* (Ornithischia; Ankylosauria) from the Lower Cretaceous of Sussex, England. Geological Magazine 133:671-682.

Blows WT. 2001. Dermal armor of the polacanthine dinosaurs. In: Carpenter K (ed) The Armored Dinosaurs. Indiana University Press, pp. 363-385.

Blows W, Honeysett K. 2014. First Valanginian *Polacanthus* *foxii* (Dinosauria, Ankylosauria) from England, from the Lower Cretaceous of Bexhill, Sussex. Proceedings of the Geologists' Association 125:233-251.

Bohlin B. 1953. Fossil reptiles from Mongolia and Kansu. Sino-Swedish Expedition Publication 37: 1-113.

deBraga M, Rieppel O. 1997. Reptile phylogeny and the interrelationships of turtles. Zoological Journal of the Linnean Society 120: 281–354.

Burns ME. 2008. Taxonomic utility of ankylosaur (Dinosauria, Ornithischia) osteoderms: *Glyptodontopelta* *mimus* Ford, 2000: a test case. Journal of Vertebrate Paleontology 28: 1102-1109.

Burns ME, Sullivan RM. 2011. A new ankylosaurid from the Upper Cretaceous Kirtland Formation, San Juan Basin, with comments on the diversity of ankylosaurids in New Mexico. New Mexico Museum of Natural History and Science Bulletin, 53: 169-178.

Burns ME, Currie PJ. 2014. External and internal structure of ankylosaur (Dinosauria, Ornithischia) osteoderms and their systematic relevance. Journal of Vertebrate Paleontology 34: 835-851.

Carpenter K. 1990. Ankylosaur systematics: example using *Panoplosaurus* and *Edmontonia*. In: Carpenter K, Currie PJ (eds) Dinosaur systematics: approaches and perspectives. Cambridge University Press, pp. 281-298.

Carpenter K. 2001. Phylogenetic analysis of the Ankylosauria. In: Carpenter K (ed.) The Armored Dinosaurs. Indiana University Press, pp. 455-483.

Carpenter K, Everhart MJ. 2007. Skull of the ankylosaur *Niobrarasaurus* *coleii* (Ankylosauria: Nodosauridae) from the Smoky Hill Chalk (Coniacian) of western Kansas. Transactions of the Kansas Academy of Science 110:1-9.

Carpenter K, Kirkland JI. 1998. Review of Lower and Middle Cretaceous ankylosaurs from North America. New Mexico Museum of Natural History and Science Bulletin 14: 249-270.

Carpenter K, Dilkes D, Weishampel DB. 1995. The dinosaurs of the Niobrara Chalk Formation (Upper Cretaceous, Kansas). Journal of Vertebrate Paleontology 15:275-297.

Carpenter K, Miles C, Cloward K. 1998. Skull of a Jurassic ankylosaur (Dinosauria). Nature 393: 782-783.

Carpenter K, Bartlett J, Bird J, Barrick R. 2008. Ankylosaurs from the Price River Quarries, Cedar Mountain Formation (Lower Cretaceous), east-central Utah. Journal of Vertebrate Paleontology, 28: 1089-1101.

Carpenter K, Kirkland JI, Burge D, Bird J. 1999. Ankylosaurs (Dinosauria: Ornithischia) of the Cedar Mountain Formation, Utah, and their stratigraphic distribution. In Gillette D (ed) Vertebrate Paleontology in Utah. Utah Geological and Mineral Survey, pp. 243-251.

Carpenter K, Kirkland JI, Burge DL, Bird J. 2001. Disarticulated skull of a new primitive ankylosaurid from the Lower Cretaceous of eastern Utah. In: Carpenter K (ed) The Armored Dinosaurs. Indiana University Press, pp. 211-238.

Chen R, Zheng W, Azuma Y, Shibata M, Lou T, Jin Q, Jin X. 2013. A new nodosaurid ankylosaur from the Chaochuan Formation of Dongyang, Zhejiang Province, China. Acta Geologica Sinica (English Edition) 87: 801-840.

Coombs WP, Deméré TA. 1996. A Late Cretaceous nodosaurid ankylosaur (Dinosauria: Ornithischia) from marine sediments of coastal California. Journal of Paleontology 70: 311-326.

Coombs WP. 1995. A new nodosaurid ankylosaur (Dinosauria: Ornithischia) from the Lower Cretaceous of Texas. Journal of Vertebrate Paleontology 15:298-312.

Coombs WP, Maryańska T. 1990. Ankylosauria. In: Weishampel DB, Dodson P, Osmólska H (eds.) The Dinosauria, 1^st^ Edition. University of California Press, pp. 456-483.

Coria RA, Salgado L. 2001. South American ankylosaurs. In: Carpenter K (ed.) The Armored Dinosaurs. Indiana University Press, pp. 159-168.

Dong Z. 1993. An ankylosaur (ornithischian dinosaur) from the Middle Jurassic of the Junggar Basin, China. Vertebrata PalAsiatica 31: 257-266.

Eaton TH. 1960. A new armored dinosaur from the Cretaceous of Kansas. The University of Kansas Paleontological Contributions Vertebrata 8:1-24.

Everhart MJ, Hamm SA. 2005. A new nodosaur specimen (Dinosauria: Nodosauridae) from the Smoky Hill Chalk (Upper Cretaceous) of western Kansas. Transactions of the Kansas Academy of Science 108:15-21.

Ford TL. 2000. A review of ankylosaur osteoderms from New Mexico and a preliminary review of ankylosaur armor. New Mexico Museum of Natural History and Science Bulletin 17: 157-176.

Ford TL, Kirkland JI. 2001. Carlsbad ankylosaur: an ankylosaurid and not a nodosaurid. In: Carpenter K (ed.) The Armored Dinosaurs. Indiana University Press, pp. 239-260.

Galton PM. 1980. Partial skeleton of *Dracopelta* *zbyszewskii* n. gen. and n. sp., an ankylosaurian dinosaur from the Upper Jurassic of Portugal. Géobios 13:451-457.

Garcia G, Pereda Suberbiola X. 2003. A new species of *Struthiosaurus* (Dinosauria: Ankylosauria) from the Upper Cretaceous of Villeveyrac (southern France). Journal of Vertebrate Paleontology 23:156-165.

Gilmore CW. 1930. On dinosaurian reptiles from the Two Medicine Formation of Montana. Proceedings of the United States National Museum 77:1-39.

Godefroit P, Pereda-Suberbiola X, Li H, Dong Z. 1999. A new species of the ankylosaurid dinosaur *Pinacosaurus* from the Late Cretaceous of Inner Mongolia (P.R. China). Bulletin de l'Institut Royal des Sciences Naturelles de Belgique, Sciences de la Terre 69(suppl.): 17-366.

Han F, Zheng W, Hu D, Xu X, Barrett PM. 2014. A new basal ankylosaurid (Dinosauria: Ornithichia) from the Lower Cretaceous Jiufotang Formation of Liaoning Province, China. PLOS ONE 9: e104551.

Heckert AB, Lucas SG. 1999. A new aetosaur (Reptilia: Archosauria) from the Upper Triassic of Texas and the phylogeny of aetosaurs. Journal of Vertebrate Paleontology 19: 50–68.

Hill RV. 2005. Integrative morphological data sets for phylogenetic analysis of Amniota: the importance of integumentary characters and increased taxonomic sampling. Systematic Biology 54: 530–547.

Hill RV, Witmer LW, Norell MA. 2003. A new specimen of *Pinacosaurus grangeri* (Dinosauria: Ornithischia) from the Late Cretaceous of Mongolia: ontogeny and phylogeny of ankylosaurs. American Museum Novitates 3395: 1-29.

Jacobs LL, Winkler DA, Murry PA, Maurice JM. 1994. A nodosaurid scuteling from the Texas shore of the Western Interior Seaway. In: Carpenter K, Hirsch KF, Horner JR (eds) Dinosaur eggs and babies. Cambridge University Press, pp. 337-346.

Killbourne B, Carpenter K. 2005. Redescription of *Gargoyleosaurus* *parkpinorum*, a polacanthid ankylosaur from the Upper Jurassic of Albany County, Wyoming. Neues Jahrbuch für Geologie und Paläontologie 237:111-160.

Kirkland JI, Alcalá L, Loewen MA, Espílez E, Mampel L, Wiersma J. 2013. The basal nodosaurid ankylosaur *Europelta* *carbonensis* n. gen., n. sp. from the Lower Cretaceous (Lower Albian) Eschucha Formation of northeastern Spain. PLOS ONE 8:e80405.

Kirkland JI. 1998. A polacanthine ankylosaur (Ornithischia: Dinosauria) from the Early Cretaceous (Barremian) of eastern Utah. New Mexico Museum of Natural History and Science Bulletin 14: 271-281.

Kirkland JI, Carpenter K. 1994. North America's first pre-Cretaceous ankylosaur (Dinosauria) from the Upper Jurassic Morrison Formation of western Colorado. BYU Geology Studies 40: 25-42.

Kirkland JI, Carpenter K, Hunt AP, Scheetz RD. 1998. Ankylosaur (Dinosauria) specimens from the Upper Jurassic Morrison Formation. Modern Geology 23: 145-177.

Lambe LM. 1919. Description of a new genus and species (*Panoplosaurus* *mirus*) of an armoured dinosaur from the Belly River Beds of Alberta. Transactions of the Royal Society of Canada, series 3 13: 39-50.

Leahey LG, Molnar RE, Carpenter K, Witmer LM, Salisbury SW. 2015. Cranial osteology of the ankylosaurian dinosaur formerly known as *Minmi* sp. (Ornithischia: Thyreophora) from the Lower Cretaceous Allaru Mudstone of Richmond, Queensland, Australia. PeerJ 3:e1475.

Lee MSY. 1997. Pareiasaur phylogeny and the origin of turtles. Zoological Journal of the Linnean Society 120: 197–280.

Lee Y-N. 1996. A new nodosaurid ankylosaur (Dinosauria: Ornithischia) from the Paw Paw Formation (Late Albian) of Texas. Journal of Vertebrate Paleontology 16:232-245.

Lü J, Ji Q, Gao Y, Li Z. 2007a. A new species of the ankylosaurid dinosaur *Crichtonsaurus* (Ankylosauridae : Ankylosauria) from the Cretaceous of Liaoning Province, China. Acta Geologica Sinica 81: 883-897.

Lü J, Jin X, Sheng Y, Li Y, Wang G, Azuma Y. 2007b. New nodosaurid dinosaur from the Late Cretaceous of Lishui, Zhejiang Province, China. Acta Geologica Sinica 81: 344-350.

Lucas FA. 1901. A new dinosaur, *Stegosaurus* *marshi*, from the Lower Cretaceous of South Dakota. Proceedings of the United States National Museum 23:591-592.

Lull RS. 1921. The Cretaceous armored dinosaur, *Nodosaurus* *textilis* Marsh. The American Journal of Science, Fifth Series 1:97-126.

Lydekker R. 1893. On the jaw of a new carnivorous dinosaur from the Oxford Clay of Peterborough. Quarterly Journal of the Geological Society 49: 284-287.

Maleev EA. 1952. [A new ankylosaur from the Upper Cretaceous of Mongolia.] Doklady Akademii Nauk, SSSR 87: 273-276. [In Russian; translation by T. and F. Jeletsky, 1956]

Mantell GA. 1833. The geology of the south-east of England. Longman Ltd., 415 p.

Marsh OC. 1889. Notice of gigantic horned Dinosauria from the Cretaceous. American Journal of Science 38:173-175.

Marsh OC. 1888. Notice of a new genus of Sauropoda and other new dinosaurs from the Potomac Formation. American Journal of Science 135:89-94.

Maryańska T. 1977. Ankylosauridae (Dinosauria) from Mongolia. Palaeontologia Polonica 37: 85-151.

Mehl MG. 1936. *Hierosaurus* *coleii*: a new aquatic dinosaur from the Niobrara Cretaceous of Kansas. Denison University Bulletin, Journal of the Scientific Laboratory 31:1-20.

Miyashita T, Arbour VM, Witmer LM, Currie PJ. 2011. The internal cranial morphology of an armoured dinosaur Euoplocephalus corroborated by X-ray computed tomographic reconstruction. Journal of Anatomy 219: 661-675.

Molnar RE. 1980. An ankylosaur (Ornithischia: Reptilia) from the Lower Cretaceous of southern Queensland. Memoirs of the Queensland Museum 20:77-87.

Molnar RE. 1996. Preliminary report on a new ankylosaur from the Early Cretaceous of Queensland, Australia. Memoirs of the Queensland Museum 39:653-668.

Molnar RE. 2001. Armor of the small ankylosaur *Minmi*. In: Carpenter K (ed) The Armored Dinosaurs. Indiana University Press, pp. 341-362.

Molnar RE, Frey E. 1987. The paravertebral elements of the Australian ankylosaur *Minmi* (Reptilia: Ornithischia, Cretaceous). Neues Jahrbuch für Geologie un Paläontologie, Abhandlungen 175:19-37.

Moodie RL. 1910. An armored dinosaur from the Cretaceous of Wyoming. The Kansas University Science Bulletin 5: 257-273.

Nopcsa FB. 1915. [The dinosaurs of the Transylvanian province in Hungary]. [Communications of the Yearbook of the Royal Hungarian Geological Imperial Institute] 23:1-24. [In Hungarian]

Ősi A. 2005. *Hungarosaurus* *tormai*, a new ankylosaur (Dinosauria) from the Upper Cretaceous of Hungary. Journal of Vertebrate Paleontology 25:370-383.

Ősi A, Makádi L. 2009. New remains of *Hungarosaurus* *tormai* (Ankylosauria, Dinosauria) from the Upper Cretaceous of Hungary: skeletal reconstruction and body mass estimation. Paläontologische Zeitschrift 83:227-245.

Ősi A, Prondvai E. 2013. Sympatry of two ankylosaurs (*Hungarosaurus* and cf. *Struthiosaurus*) in the Santonian of Hungary. Cretaceous Research 44:58-63.

Ősi A, Pereda Suberbiola X, Földes T. 2014. Partial skull and endocranial cast of the ankylosaurian dinosaur *Hungarosaurus* from the Late Cretaceous of Hungary: implications for locomotion. Palaeontologia Electronica 17:1-18.

Ostrom JH. 1970. Stratigraphy and Paleontology of the Cloverly Formation (Lower Cretaceous) of the Bighorn Basin Area, Wyoming and Montana. Peabody Museum of Natural History, Yale University, Bulletin 35, 234p.

Parish JC, Barrett PM. 2004. A reappraisal of the ornithischian dinosaur *Amtosaurus magnus* Kurzanov and Tumanova 1978, with comments on the status of *A. archibaldi* Averianov 2002. Canadian Journal of Earth Sciences 41: 299-306.

Parsons WL, Parsons KM. 2009. A new ankylosaur (Dinosauria: Ankylosauria) from the Lower Cretaceous Cloverly Formation of central Montana. Canadian Journal of Earth Sciences 46: 721-738.

Pereda-Suberbiola J. 1993. *Hylaeosaurus*, *Polacanthus*, and the systematics and stratigraphy of Wealden armoured dinosaurs. Geological Magazine 130:767-781.

Pereda-Suberbiola X, Galton PM. 1994. A revision of the cranial features of the dinosaur *Struthiosaurus* *austriacus* Bunzel (Ornithischia: Ankylosauria) from the Late Cretaceous of Europe. Neues Jahrbuch für Geologie un Paläontologie, Abhandlungen 191:173-200.

Pereda Suberbiola X, Barrett PM. 1999. A systematic review of ankylosaurian dinosaur remains from the Albian-Cenomanian of England. The Palaeontological Association, Special Papers in Palaeontology 60:177-288.

Pereda Suberbiola X, Galton PM. 2001. Reappraisal of the nodosaurid ankylosaur *Struthiosaurus* *austriacus* Bunzel from the Upper Cretaceous Gosau Beds of Austria. In: Carpenter K. (ed) The Armored Dinosaurs. Indiana University Press, pp. 173-210.

Salgado L, Gasparini Z. 2006. Reappraisal of an ankylosaurian dinosaur from the Upper Cretaceous of James Ross Island (Antarctica). Geodiversitas 28:119-135.

Sereno PC. 1986. Phylogeny of the bird-hipped dinosaurs (order Ornithischia). National Geographic Research 2: 234–256.

Sereno PC. 1999. The evolution of dinosaurs. Science 284: 2137-2147.

Sternberg CM. 1928. A new armored dinosaur from the Edmonton Formation of Alberta. Transactions of the Royal Society of Canada series 3 22:93-106.

Sullivan RM. 1999. *Nodocephalosaurus* *kirtlandensis*, gen. et sp nov., a new ankylosaurid dinosaur (Ornithischia: Ankylosauria) from the Upper Cretaceous Kirtland Formation (Upper Campanian), San Juan Basin, New Mexico. Journal of Vertebrate Paleontology 19: 126-139.

Thompson RS, Parish JC, Maidment SCR, Barrett PM. 2012. Phylogeny of the ankylosaurian dinosaurs (Ornithischia: Thyreophora). Journal of Systematic Palaeontology 10: 301-312.

Tumanova TA. 1983. [The first ankylosaur from the Lower Cretaceous of Mongolia.] Trudy Sovmestnoi Sovestsko-Mongol’skoi Paleontologicheskoi Expeditsii 24: 110-120. [In Russian, translation by R. Welch]

Tumanova TA. 1983. [The first ankylosaur from the Lower Cretaceous of Mongolia.] Trudy Sovmestnoi Sovestsko-Mongol’skoi Paleontologicheskoi Expeditsii 24: 110-120. [In Russian, translation by R. Welch]

Vickaryous MK, Maryańska T, Weishampel DB. 2004. Ankylosauria. In Weishampel DB, Dodson P, Osmólska H (eds.) The Dinosauria, 2^nd^ Edition. University of California Press, pp. 363-392.

Vickaryous MK, Russell AP, Currie PJ, Zhao X-J. 2001. A new ankylosaurid (Dinosauria: Ankylosauria) from the Lower Cretaceous of China, with comments on ankylosaurian relationships. Canadian Journal of Earth Sciences 38: 1767-1780.

Williston SW. 1905. A new armored dinosaur from the Upper Cretaceous of Wyoming. Science 22:503-504.

Witmer LM, Ridgely RC. 2008. The paranasal air sinuses of predatory and armored dinosaurs (Archosauria: Theropoda and Ankylosauria) and their contribution to cephalic structure. Anatomical Record 291: 1362-1388.

Xu X, Wang X-L, You H-L. 2001. A juvenile ankylosaur from China. Naturwissenschaften 88: 297-300.

Yang J-T, You H-L, Li D-Q, Kong D-L. 2013. First discovery of polacanthine ankylosaur dinosaur in Asia. Vertebrata PalAsiatica 7: 17-30. [In Chinese, with English abstract]
